# p62/SQSTM1 enhances breast cancer stem-like properties by stabilizing MYC mRNA

**DOI:** 10.1038/onc.2016.202

**Published:** 2016-06-27

**Authors:** L-Z Xu, S-S Li, W Zhou, Z-J Kang, Q-X Zhang, M Kamran, J Xu, D-P Liang, C-L Wang, Z-J Hou, X-B Wan, H-J Wang, E W-F Lam, Z-W Zhao, Q Liu

**Affiliations:** 1 The Second Affiliated Hospital, Institute of Cancer Stem Cell, Dalian Medical University, Dalian, China; 2 State Key Laboratory of Oncology in South China, Collaborative Innovation Center for Cancer Medicine, Sun Yat-sen University, Guangzhou, China; 3 Department of Oncology, The First Affiliated Hospital of Guangdong Pharmaceutical University, Guangzhou, China; 4 Department of Radiation Oncology, The Sixth Affiliated Hospital, Sun Yat-sen University, Guangzhou, China; 5 Department of Breast Surgery, The First Affiliated Hospital, Dalian Medical University, Dalian, China; 6 Department of Surgery and Cancer, Imperial College London, London, UK; 7 Department of Breast Surgery, The Second Affiliated Hospital, Dalian Medical University, Dalian, China

## Abstract

Aberrant p62 overexpression has been implicated in breast cancer development. Here, we found that p62 expression was elevated in breast cancer stem cells (BCSCs), including CD44^+^CD24^−^ fractions, mammospheres, ALDH1^+^ populations and side population cells. Indeed, short-hairpin RNA (shRNA)-mediated knockdown of p62 impaired breast cancer cells from self-renewing under anchorage-independent conditions, whereas ectopic overexpression of p62 enhanced the self-renewal ability of breast cancer cells *in vitro*. Genetic depletion of p62 robustly inhibited tumor-initiating frequencies, as well as growth rates of BCSC-derived tumor xenografts in immunodeficient mice. Consistently, immunohistochemical analysis of clinical breast tumor tissues showed that high p62 expression levels were linked to poorer clinical outcome. Further gene expression profiling analysis revealed that p62 was positively correlated with MYC expression level, which mediated the function of p62 in promoting breast cancer stem-like properties. MYC mRNA level was reduced upon p62 deletion by siRNA and increased with p62 overexpression in breast cancer cells, suggesting that p62 positively regulated MYC mRNA. Interestingly, p62 did not transactivate *MYC* promoter. Instead, p62 delayed the degradation of MYC mRNA by repressing the expression of let-7a and let-7b, thus promoting MYC mRNA stabilization at the post-transcriptional level. Consistently, let-7a and let-7b mimics attenuated p62-mediated MYC mRNA stabilization. Together, these findings unveiled a previously unappreciated role of p62 in the regulation of BCSCs, assigning p62 as a promising therapeutic target for breast cancer treatments.

## Introduction

As a highly heterogeneous malignancy, breast cancer is the leading cause of female cancer-related deaths.^1, 2, 3^ Although the mortality rate has drastically dropped as a result of improved early diagnostic methods and therapeutic advancements,^4^ a significant portion of patients eventually suffer from tumor relapse and develop drug resistance. Accumulating evidence demonstrates that breast cancer stem cells (BCSCs), a tumor subpopulation which is critical for breast cancer relapse and drug resistance,^4, 5^ possess indefinite potential for self-renewal that drives tumorigenesis.^6^ For example, breast carcinomas with high ALDH1^+^ activity are capable of self-renewal and generating tumors that recapitulate the heterogeneity of the parental tumor.^7^ In addition, identification and isolation of cancer stem cells from a variety of malignancies provides a powerful tool to investigate the tumorigenic process.^8, 9, 10, 11, 12, 13, 14, 15^ Recent studies showed that genes related to the epithelial-to-mesenchymal process, such as *Slug*, *Gli-2*, *ZEB-1* and *ZEB-2*, are enriched in the CD44^+^CD24^−/low^ subpopulation.^16^ Compared with CD24^+^ cells, TGF-β signaling activities, as well as expression of TGF-β1 receptor TGF-BR II, are elevated in CD44^+^ cells derived from breast cancer tissues.^17^ CD44^+^CD24^−/low^ subpopulation tumor cells are significantly associated with lymph-node involvement, estrogen receptor and progestogen receptor status and a shorter cumulative disease-free survival (DFS) and overall survival (OS).^18^ Consistently, tumor cells derived from the CD44^+^CD24^−/low^ESA^+^ subpopulation preferentially survive chemotherapy.^19^ CD24^−/low^/CD44^+^ BCSCs are also more resistant to fractionated doses of irradiation, providing explanation for the accelerated repopulation of tumor cells observed during intervals in radiotherapy.^20^ Our recent work has shown that epirubicin-resistant MCF-7 (EpiR-MCF-7) cells contain a higher percentage of CD44^+^CD24^−/low^ subpopulation and abundance of self-renewal gene expression (for example, *CTNNB1*, *MYC*, *SOX2*, *POU5F1* and *NANOG*).^21^ However, due to the high heterogeneity and dynamic progression of BCSCs, no effective strategies have hitherto been developed to eliminate BCSCs.

The signaling protein p62 (also termed SQSTM1, ZIP3, A170) is originally identified as a cytosolic 62-kDa protein, which can bind to Src homology 2 (SH2) domain of p56^lck^.^22^ Further studies reveal that p62 contains several important functional domains, which mediate the cross-talk with a number of signaling molecules to regulate the activities of downstream effectors, such as NRF2,^23^ NF-κB^24, 25^ and mTOR.^26^ Additionally, p62 is required during autophagic process owing to its ability to bind the ubiquitinated autophagic substrates through the UBA domain, and the ability to interact with LC3 through the LIR domain.^27^ Recent studies indicate that aberrant p62 overexpression is implicated in numerous cancer types,^28, 29, 30, 31, 32^ including breast cancer.^33, 34, 35^ For example, accumulation of p62 promotes hepatocellular carcinoma development through NRF2 stabilization, leading to persistent transcriptional activation of antioxidant target genes, such as *Nqo1* and *Gstm1.*^36^ 5q copy number gains-driven overexpression of p62 mediates resistance to redox stress in kidney cancer.^32^ In breast cancer, p62 is significantly correlated with advanced clinical stages as well as a higher proportion of positive lymph nodes and lymphovascular invasion.^35^ High p62 expression is also associated with positive EGF receptor, HER2, HER3, HER4 and distant metastasis in breast cancer showing aggressive features.^34^ These findings endow p62 with a potential oncogenic role in tumor progression. However, the detailed mechanisms for p62-mediated breast cancer initiation and progression, especially a role of p62 in promoting breast cancer stem-like properties, are still poorly characterized. In this study, we set out to investigate the potential role of p62 in the regulation of breast cancer stem-like properties and the mechanism involved.

## Results

### p62 expression is elevated in BCSC-enriched populations

**Figure 1:**
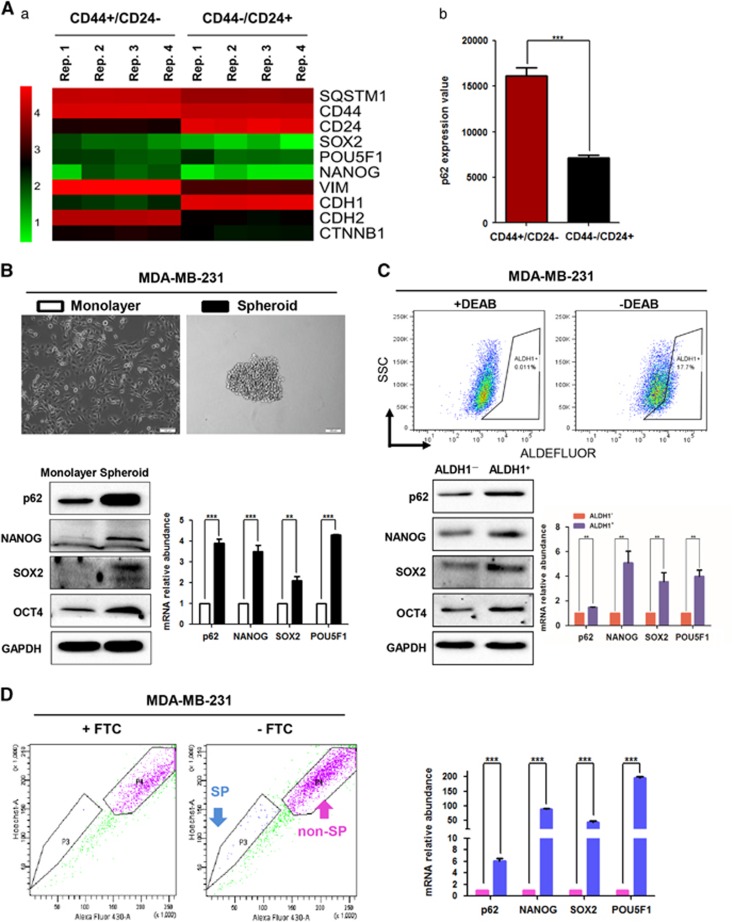
Expression of p62 is elevated in BCSC-enriched populations. (**A**) (a) Heat map displayed the expression signature of candidate genes using genome mRNA expression profiling data (GSE15192) from GEO database. (b) Column graph represented the comparison of p62 expression values between CD44^+^/CD24^−^ and CD44^−^/CD24^+^ subpopulations of MCF-10A cells. ***P<0.001, two-tailed Student's *t*-tests. BCSC-enriched populations were enriched for sphere formation assay (**B**), ALDH1-positive sorting assay (**C**) and side population assay (**D**), respectively. Expression of candidate genes (*p62*, *SOX2*, *NANOG*, *POU5F1*) and their related proteins were analyzed by RT-qPCR and western blot, respectively. **P<0.01, ***P<0.001, two-tailed Student's *t*-tests. Error bars represented mean±s.d.

The CD44^+^/CD24^−^ subpopulations of normal and cancerous breast epithelial cells are suggested to display stem-cell properties.^8^ In an effort to explore the relationship between p62 and breast cancer stem-like properties, we first analyzed the gene expression patterns of CD44^+^/CD24^−^ and CD44^−^/CD24^+^ subpopulations of the MCF-10A breast epithelial cell line with basal cell phenotype (GSE15192 from the PubMed GEO database).^16^ As shown in the heat map of Figure 1A(a), genes that mediated stem-like properties, such as *SOX2*, *POU5F1* (also known as OCT4) and *NANOG*, were found to elevate in the CD44^+^/CD24^−^ subpopulation. Notably, p62 (also known as SQSTM1) expression value of the CD44^+^/CD24^−^ subpopulation was significantly higher than that of CD44^−^/CD24^+^ subpopulation (Figure 1A(b)).

Next, we conducted mammosphere formation, ALDH1^+^ and side population (SP) sorting assays to enrich for BCSCs for subsequent mRNA and protein expression analysis. SOX2, POU5F1 and NANOG were employed as markers for stem-like properties of the enriched populations. Compared with cells grown in monolayer cultures, spheroids isolated from MDA-MB-231 cells displayed higher mRNA and protein levels of p62 expression, associated with higher expression levels of SOX2, POU5F1 and NANOG (Figure 1B). In addition, spheroids isolated from SUM149 cells showed higher p62 mRNA expression levels compared with monolayer cells (Supplementary Figure 1A). ALDH1-positive sorting assay indicated that ALDH1^+^ cells also exhibited much higher p62 mRNA and protein expression levels than ALDH1^−^ cells (Figure 1C). Moreover, increased p62 mRNA expression was also observed in the SP compared with the non-SP isolated from MDA-MB-231 cells (Figure 1D). Together, these observations indicated that p62 was elevated in the BCSC-enriched populations, suggesting that p62 has a key role in promoting breast cancer stem-like properties.

### p62 is an important mediator of stem-like properties for breast cancer cells *in vitro*

**Figure 2:**
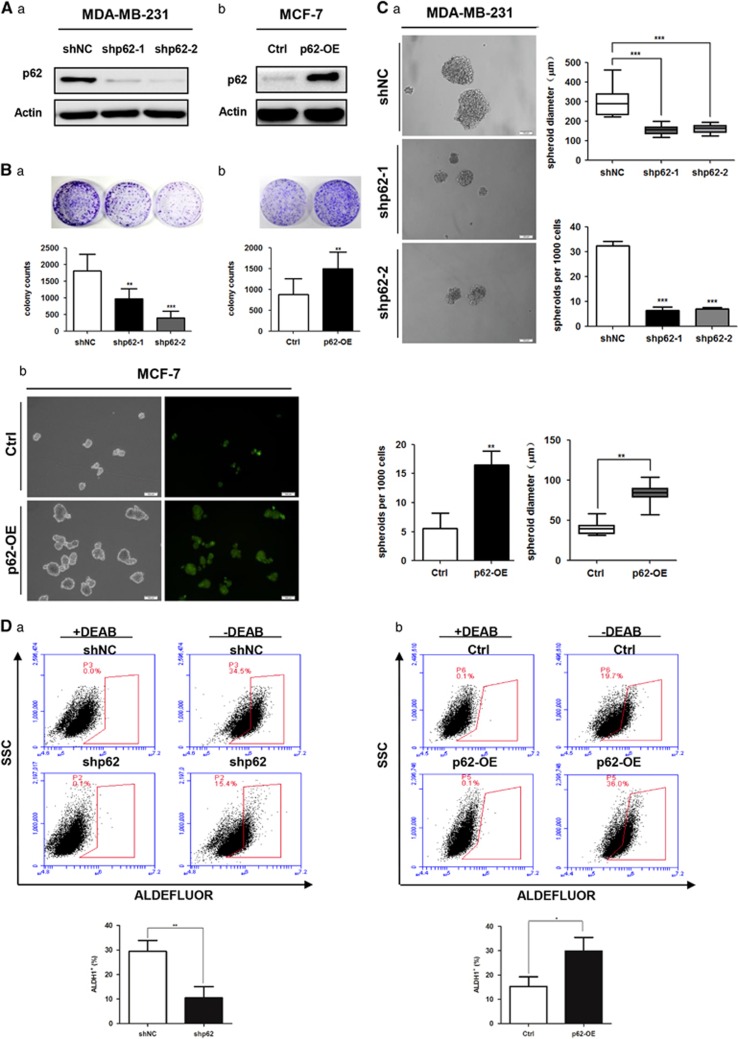
p62 is essential for breast cancer cells to maintain stem-like properties *in vitro*. (**A**) Efficiencies for knockdown or overexpression of p62 were tested by western blot analysis. (**B**) Comparison of plate colony formation numbers and (**C**) mammosphere-forming abilities and ALDH1-positive populations (**D**) were analyzed following p62 interference. *P<0.05, **P<0.01, ***P<0.001. Error bars represented mean±s.d. Scale bars, 100 μm.

To assess the contribution of p62 in promoting breast cancer stem-like properties, we used short-hairpin RNA (shRNA) to mediate p62 suppression in MDA-MB-231 and SUM149 cells with relatively higher endogenous p62 expression levels, and lentivirus to facilitate ectopic overexpression of p62 in MCF-7 cells which displayed lower p62 levels (Supplementary Figure 2). The efficiencies of p62 depletion and overexpression were then assessed by western blotting. As shown in Figure 2A and Supplementary Figure 1B, p62 was effectively depleted by two different shRNAs in MDA-MB-231 and SUM149 cells, and significantly induced by lentivirus transduction in MCF-7 cells.

Next, we performed plate colony formation, mammosphere formation and ALDH1-positive sorting assays to investigate the role of p62 in promoting self-renewal abilities, a key characteristic of BCSCs. The colony numbers (up to 14 days) on regulate plates were markedly decreased following the depletion of endogenous p62 by shRNA in both the MDA-MB-231 and the SUM149 cells (Figure 2B(a) and Supplementary Figure 1C). Moreover, the sphere formation efficiency was dramatically reduced upon p62 depletion, as indicated by a decrease in both spheroid numbers and diameters (Figure 2C(a) and Supplementary Figure 1D). Conversely, both the colony number and the sphere formation ability were enhanced by p62 overexpression in MCF-7 cells (Figures 2B(b) and 2C(b)). Interestingly, the ALDH1^+^ population was markedly decreased following the depletion of p62 and increased by p62 overexpression in MDA-MB-231 cells (Figures 2D(a) and (b)). In addition, we also conducted shRNA-mediated p62 knockdown in MDA-MB-231 cells and lentivirus-mediated p62 overexpression in MCF-7 cells for both RT-qPCR and western blot analysis. As shown in Supplementary Figure 3, p62 knockdown reduced both the mRNA and the protein levels of NANOG, SOX2 and POU5F1 (OCT4), whereas p62 upregulation promoted the expression of these stemness markers. Together, these data implied that p62 is necessary for promoting breast cancer stem-like properties *in vitro*.

### Depletion of p62 abrogated the tumor-initiating frequencies and growth rates of BCSC-derived tumor xenografts *in vivo*

**Figure 3:**
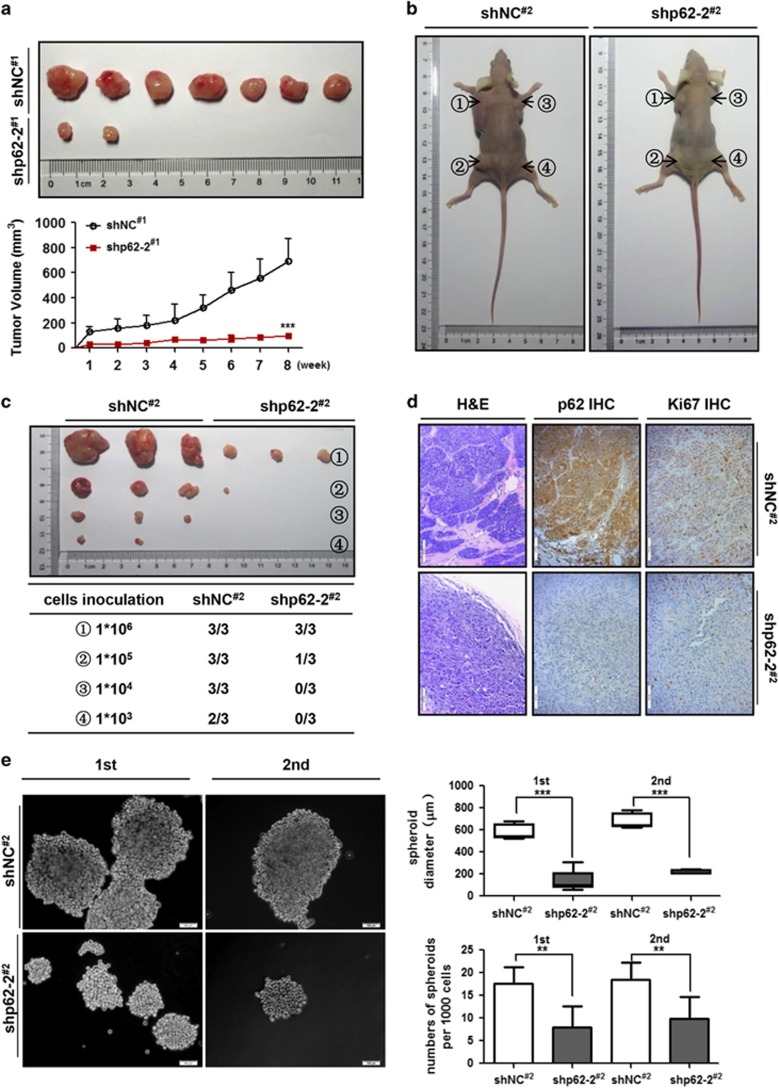
Suppression of p62 impairs the tumor-initiating ability of breast cancer cells *in vivo*. (**a**) Immunodeficient mice (*n*=5, 2 sites per mouse) were subcutaneously inoculated with equal number of single cells (1 × 10^6^ cells per mouse). Tumor xenografts were monitored for 8 weeks. Tumor volumes were monitored as described in Materials and methods. (**b**) Limiting diluted numbers of isolated tumor cells were subcutaneously inoculated into immunodeficient mice (*n*=3) at the indicated sites. (**c**) Serial transplantation frequencies were analyzed after 8 weeks. (**d**) H&E and IHC staining of xenografted tumor tissues. Scale bars, 100 μm. (**e**) Isolated single tumor cells from tumor xenografts were subjected to sphere formation assay. Both first and second mammosphere-forming abilities were compared. **P<0.01, ***P<0.001. Error bars represented mean±s.d. Scale bars, 100 μm.

Given that p62 suppression diminished BCSC properties *in vitro*, we then examined whether p62 knockdown would affect the tumor-initiating potential of breast cancer cells. To this end, we generated MDA-MB-231-shp62-2 stable cells with good knockdown efficiency (Figure 2A(a)). An equal number of p62 knockdown MDA-MB-231 cells (1 × 10^6^ cells per mouse) were subcutaneously injected into 4- to 6-week-old BALB/C (nu/nu) female nude mice (5 mice in each group, 2 sites for each mouse). Tumor xenografts were then monitored every week after inoculation. Mice were killed after 8 weeks and tumor formation incidences were analyzed. As shown in Figure 3a, mice inoculated with the shp62-2 cells had a lower tumor incidence (2/10) and tumors derived from shp62-2 stable cells (referred as shp62-2^#1^) also grew at a significantly reduced rate compared with shNC cells (referred as shNC^#1^, 7/10).

Next, limiting dilution assay was employed to evaluate whether depletion of p62 would impair the tumor-forming abilities of xenograft-derived tumor cells. Single cells were isolated from shNC^#1^ and shp62-2^#1^ xenografts by enzyme digestion, respectively. Then, equal amounts of cells (1 × 10^6^, 1 × 10^5^, 1 × 10^4^ and 1 × 10^3^) were subcutaneously inoculated into four different sites in each mouse (*n*=3) (Figure 3b) and monitored for 8 weeks. Notably, shNC cells (referred as shNC^#2^) formed tumors effectively at the dilutions of 1 × 10^6^, 1 × 10^5^ and 1 × 10^4^ (100%), with a relatively lower incidence (66.7%, 2/3 sites) at the dilution of 1 × 10^3^. The tumor formation frequencies of shp62-2 cells (referred as shp62-2^#2^) at each dilution (1 × 10^6^, 1 × 10^5^, 1 × 10^4^ and 1 × 10^3^) within the same time frame were 100, 33.3, 0 and 0%, respectively (Figure 3c). These results demonstrated that compared with control cells, depletion of p62 gave rise to secondary tumor xenografts with decreased frequencies, along with reduced tumor volumes.

Meanwhile, H&E morphological detection showed enhanced accumulation of fibroblastic tissues and massive neovascularization in shNC^#2^ tumors but not in shp62-2^#2^ tumors (Figure 3d). Immunohistochemistry (IHC) analysis of shNC^#2^ tumors revealed abundant p62 protein resided within the cytoplasm and only occasionally in the nucleus, along with massive Ki67-positive staining (62.5±12.5%) (Figure 3d and Supplementary Figure 4). In contrast, suppression of p62 resulted in a dramatic reduction in Ki67-positive staining in the shp62-2^#2^ tumors (6.0±1.0%) (Figure 3d and Supplementary Figure 4). In addition, single cells extracted from secondary tumor xenografts were subjected to mammosphere formation assay to assess for their self-renewal abilities. As shown in Figure 3e, both first and second generation of shp62 cells (isolated from shp62-2^#2^ xenografts) displayed impaired mammosphere-forming efficiencies, as indicated by their significantly decreased spheroid numbers and diameters.

To further confirm that inhibition of p62 in stem-cell subpopulation can suppress tumor-initiating ability, we first performed ALDH1 sorting assay by using MDA-MB-231-shNC and shp62 stably transfected cells before injecting into NOD/SCID mice for xenograft tumor experiments (Supplementary Figure 5a). The ALDH1^+^ cells in shp62 group exhibited lower p62 mRNA expression level than in shNC group, along with lower expression of NANOG, SOX2 and POU5F1 (Supplementary Figure 5b). Then, two group with equal amounts of ALDH1^+^ cells (5 × 10^4^) were subcutaneously injected into NOD/SCID mice (*n*=6). Mice were killed after 6 weeks and tumor-initiating frequencies were analyzed. As shown in Supplementary Figure 5c and d, compared with shNC group (6/6), mice inoculated with the shp62 cells exhibited a lower tumor incidence (5/6), along with reduced tumor volumes. Collectively, these data showed that depletion of p62 abrogated the tumor-initiating efficiency and growth of BCSC-derived tumor xenografts *in vivo*, supporting a critical role of p62 in the promotion of breast cancer stem-like properties.

### High p62 expression is associated with poorer prognosis in breast cancer

**Figure 4:**
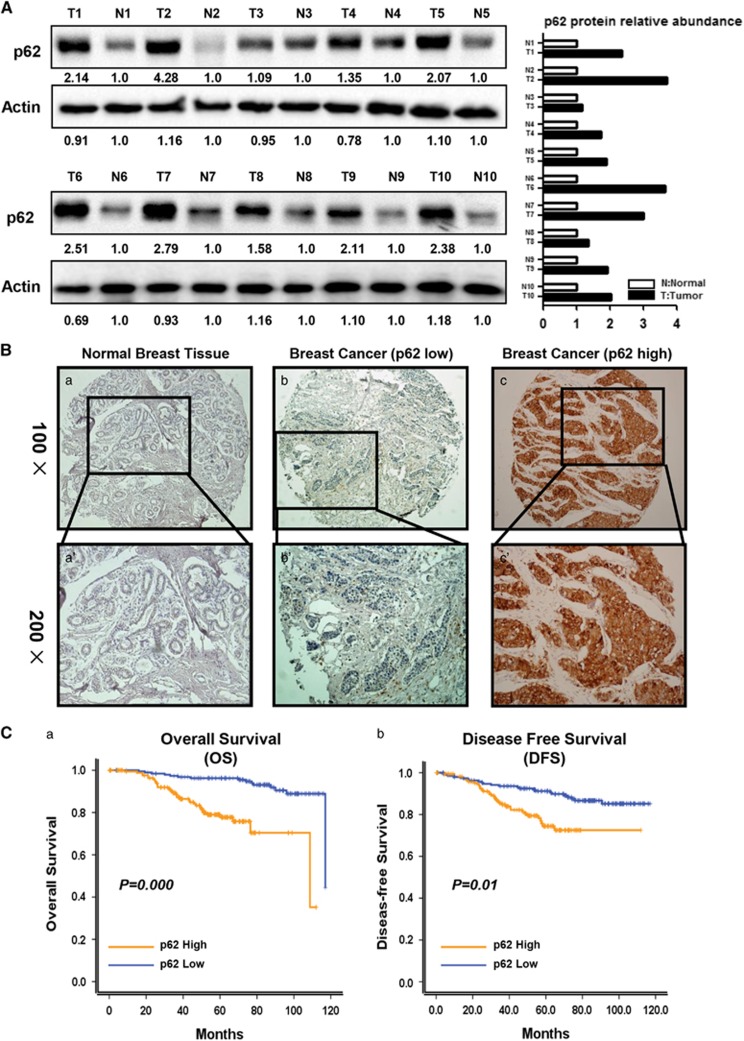
High p62 expression levels indicate poor clinical outcome in breast cancer patients. (**A**) Ten pairs of breast tumors (T) and adjacent normal tissues (N) were subjected to western blot analysis. Fold of p62 expression was normalized to adjacent normal tissue in each pair. (**B**) Representative images of negative, low and high levels of p62 staining, which showed no, few and much visible cellular brown-yellowish granules, respectively (a–c, × 100). The indicated fields of (a–c) are magnified to show the clear staining granules (a'–c', × 200). (**C**) Cumulative survival probabilities (a, OS and b, DFS) were calculated through the Kaplan–Meier method (*n*=369) based on p62 expression. Survival rates were compared by log-rank test.

To evaluate the clinical relevance of p62-mediated stem-like properties, we first collected 10 pairs of breast cancer and adjacent normal tissues, and conducted western blot analysis of p62 expression levels. The results showed that all breast cancer tissues displayed higher p62 expression levels at varying degrees when compared with adjacent normal tissues (from 1.09-fold to 4.28-fold, normalized to Actin) (Figure 4A). In addition, we also examined the p62 expression in a total of 369 breast cancer patients. (The median age is 48 years old.) IHC staining was executed to evaluate the p62 protein expression levels. Compared with normal breast tissues (employed as a negative control), the p62 protein expression was significantly higher in breast cancer tissues. (Representative images were shown in Figure 4B.) The relationship between p62 protein expression and clinicopathological parameters of breast cancer patients was summarized in Table 1. High p62 protein expression was significantly correlated with clinical stage (*P*=0.011) and node stage (*P*=0.004). In comparison, no significant correlations were discovered between p62 protein expression and other clinicopathological characteristics.

Moreover, the Kaplan–Meier survival analysis demonstrated that high level of p62 was a strong indicator for an inferior OS and DFS (Figure 4C, *P-*values were 0.000 and 0.001, respectively) in our patient cohort, suggesting a significantly unfavorable prognosis and shorter life span. In multivariate analysis, p62 was also a significant predictor for poor OS (*P*=0.000, Hazard ratio 4.669, 95% CI from 2.313 to 9.426, Table 2) and poor DFS (*P*=0.014, Hazard ratio 2.040, 95% CI from 1.156 to 3.602, Table 2). Thus, these data suggested that p62 can be a potential independent prognostic factor for breast cancer, with high p62 expression associated with poor diagnosis and prognosis.

### MYC is necessary for the maintenance of p62-mediated stem-like properties in breast cancer

**Figure 5:**
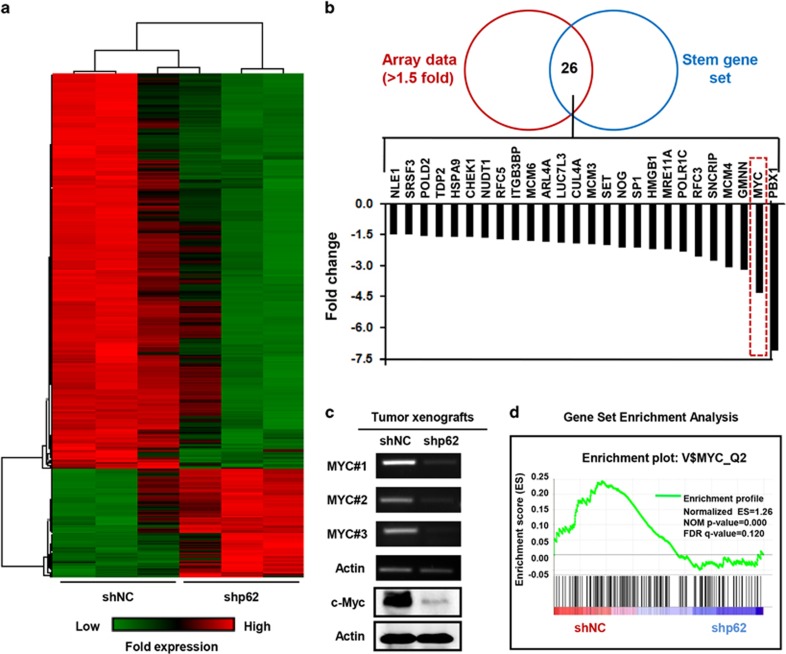
p62 is positively correlated with MYC expression level in xenografted tumor tissues. (**a**) An unbiased genome mRNA expression profiling heat map between shNC^#2^ and shp62-2^#2^ xenografted tumor tissues (analyzed from three independent tests). (**b**) Comparison of array data (>1.5-fold) with one stem gene set. Common genes were listed in the column graph according to their fold changes. (**c**) Validation of MYC gene expression by RT–PCR and western blot analysis in the paired xenografted tissues. (**d**) GSEA of MYC downstream target genes (false discovery rate (FDR)⩽0.25 and *P*<0.05 were considered significant).

In an effort to investigate the downstream target for p62-mediated stem-like properties in breast cancer, we extracted total RNA from both shNC^#2^ and shp62-2^#2^ tumor xenografts, generated the global gene expression profile by using Affymetrix Human Primeview (analyzed from independent triplicates) (Figure 5a), and then compared the array data (>1.5-fold) with one stem-cell gene set.^37^ As shown in Figure 5b, a series of stemness-associated genes showed declined expression levels in shp62-2^#2^ tumor xenografts, including the *MYC* gene (−4.389-fold), which was recently revealed to be a key factor in breast cancer stemness.^38, 39, 40^ We thus hypothesized that MYC might act as a downstream target for p62-mediated breast cancer stem-like properties. To test this conjecture, we first validated the array data by employing RT–PCR and western blot analysis. The results confirmed that MYC expression was robustly inhibited following suppression of p62 (Figure 5c). Meanwhile, considering that c-Myc protein, encoded by the *MYC* gene, is a multifunctional transcription factor that regulates transcription of various target genes, we performed the gene set enrichment analysis (GSEA) of the expression profile and found that suppression of p62 significantly diminished the enrichment of genes that are regulated by c-Myc (Figure 5d and Supplementary Figures 6a and b), indicating a reduction in c-Myc transcriptional activity.

**Figure 6:**
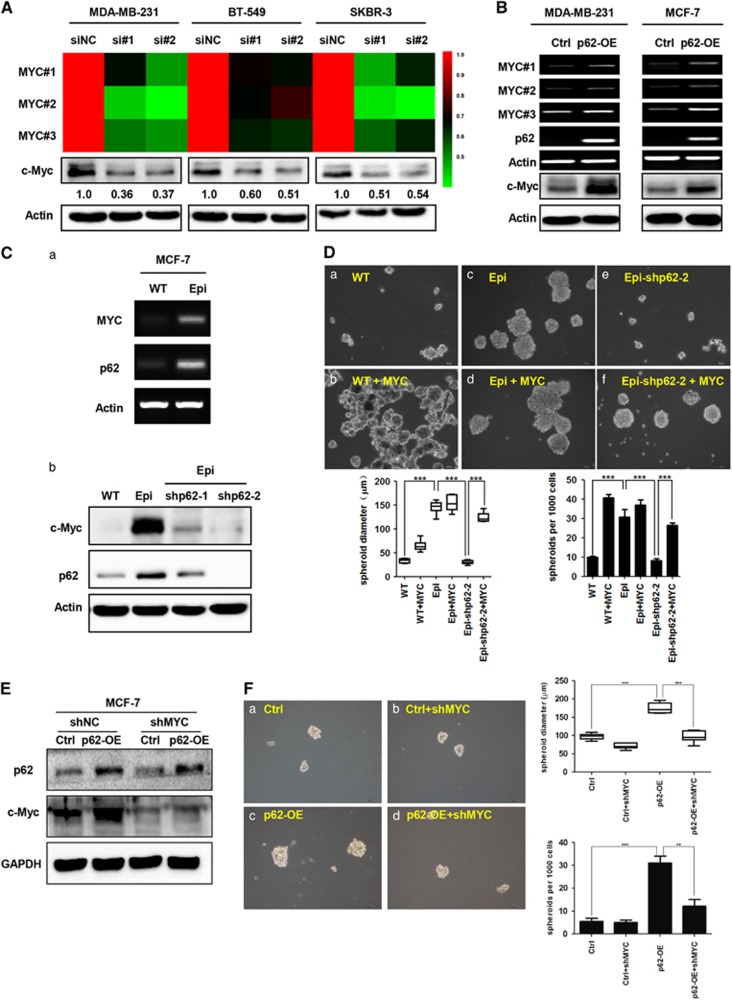
MYC acts as a functional effector for p62-mediated breast cancer stem-like properties. Validations of MYC mRNA and protein levels were examined following p62 transient knockdown (**A**) or overexpression (**B**). Transient knockdown was mediated by two different RNAi sequences (si#1, si#2). MYC mRNA abundance was detected by three different primers (MYC#1, #2, #3). Heat map displayed MYC mRNA levels from three independent RT-qPCR tests. MYC expression levels (c-Myc) were assessed by western blot and normalized to Actin. (**C**) Both p62 and MYC expression levels were measured by RT–PCR (a) and western blot (b) assays in wild-type MCF-7 (WT) and epirubicin-resistant MCF-7 (Epi) cells. Efficiencies of shRNA-mediated suppression of p62 were examined by western blot analysis (b). (**D**) Mammosphere-forming abilities were compared. ***P<0.001. Scale bars, 50 μm. (**E**) shRNA-mediated MYC suppression in both MCF-7-Ctrl and MCF-7-p62-OE cells. p62 and MYC expression levels were assessed by western blot. And mammosphere-forming abilities were compared. **P<0.01; ***P<0.001. Scale bars, 100 μm, (**F**).

As a result, we examined the correlation between p62 and MYC in breast cancer cell lines *in vitro*. Two different RNAi sequences of p62 were applied to transiently knockdown p62 in MDA-MB-231, BT-549 and SKBR-3 breast carcinoma cells. Seventy-two hours after transfection, RT-qPCR (three pairs of primers designed for MYC) and western blot assays were conducted to evaluate the mRNA and protein levels of MYC. As shown in Figure 6A, both RNAi sequences of p62 decreased the MYC mRNA and protein levels in the three cell lines. Conversely, overexpression of p62 in MDA-MB-231 and MCF-7 cell lines enhanced both the mRNA and protein levels of MYC (Figure 6B). These results confirmed that p62 is positively correlated with MYC mRNA levels in breast cancer cells.

We previously reported that epirubicin-resistant MCF-7 (EpiR-MCF-7) cells displayed abundant stem-like properties compared with their wild-type counterparts (WT-MCF-7).^21^ In this study, we found that EpiR-MCF-7 cells exhibited enhanced mammosphere-forming capacity (Figures 6D(a) and (c)). Importantly, we observed that both p62 and MYC were elevated at the mRNA and protein levels in EpiR-MCF-7 cells (Figures 6C(a) and (b)). Both shRNA-mediated knockdown of p62 caused a reduction in MYC expression in EpiR-MCF-7 cells (Figure 6C(b)), as well as a decreased ability to form mammospheres (Figures 6D(c) and (e)).

To further confirm a role of MYC for the p62-mediated stem-like properties in breast cancer, we conducted lentivirus-mediated MYC ectopic overexpression in both WT-MCF-7 and EpiR-MCF-7 cells. In accordance with previous studies, overexpression of MYC resulted in a significant increase in the mammosphere-forming ability in WT-MCF-7 cells (Figure 6D(b)). Critically, re-constitution of MYC expression in EpiR-MCF-7-shp62-2 cells abolished the reduced mammosphere-forming ability (Figure 6D(f)). In addition, we conducted shRNA-mediated MYC suppression in both MCF-7-Ctrl and MCF-7-p62-OE cells. Notably, overexpression of p62 caused an increment in MYC expression in MCF-7 cells, along with an increased ability to form mammospheres (Figures 6E, 6F(a) and (c)). Furthermore, knockdown of MYC in MCF-7-p62-OE cells attenuated the increased mammosphere-forming ability (Figure 6F(d)). Taken together, these results indicated that MYC is necessary for the p62-promoted stem-like properties in breast cancer.

### p62 promotes MYC mRNA stability at the post-transcriptional level, rather than influencing its promoter activity

**Figure 7:**
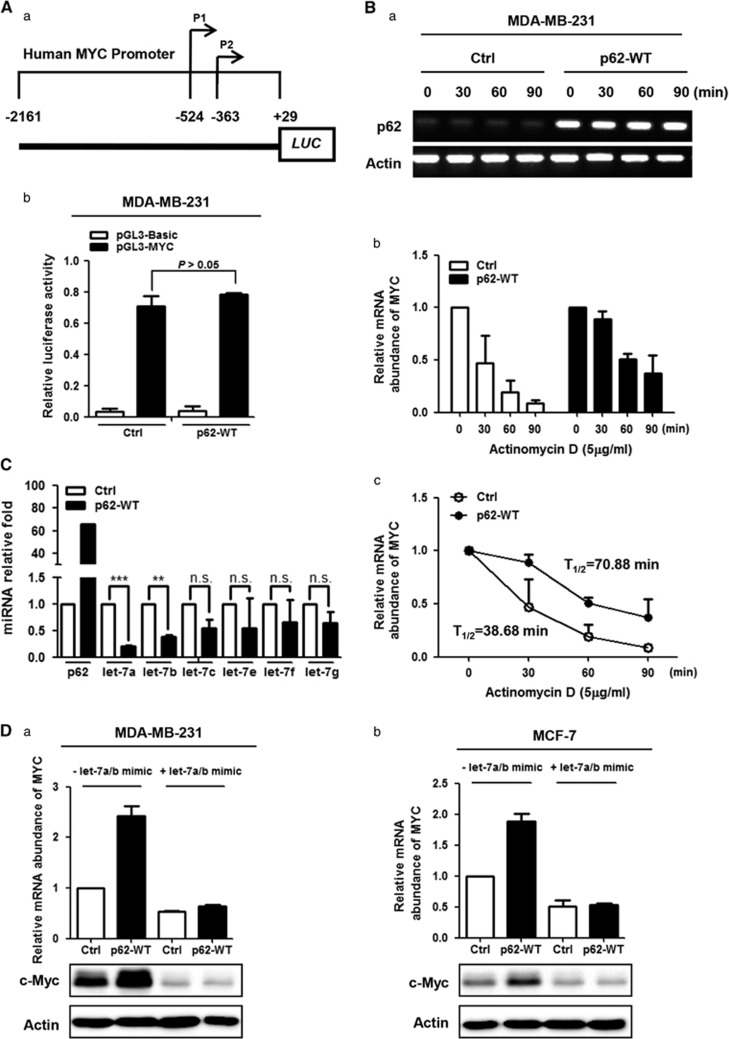
p62 promotes MYC mRNA stability after transcription. (**A**) Full length of *MYC* promoter sequence was cloned and inserted into pGL3-Basic plasmid (a), and dual-luciferase assay was performed in MDA-MB-231 cells as described in Supplementary Materials and Methods (b). Transcription activity was calculated as the ratio of Firefly luciferase activity (reporter) vs Renilla luciferase activity (control). Error bars represented mean±s.d. Comparison was analyzed by two-tailed Student's *t*-tests. (**B**) MDA-MB-231 cells were transiently transfected with wild-type (WT) p62 construct for 48 h. Actinomycin D (ActD, 5 μg/ml) was added for indicated time points (0, 30, 60 and 90 min) before harvest. MYC mRNA levels were assessed by RT-qPCR analysis. (a) Transfection efficiencies of p62 were determined by RT–PCR analysis. (b) Relative fold of expression was calculated by normalizing mRNA level to that at 0 min (without ActD treatment) for both control (Ctrl) and p62-overexpressed (p62-WT) groups. Results were representative of three independent experiments. Error bars represented mean±s.d. (c) Half-life of mRNA for each group was predicted according to fold described in (b). (**C**) Expression of the let-7 cluster was analyzed by RT-qPCR assay (RNU6-2 was used as an internal control). Error bars represented mean±s.d. Comparison was analyzed by two-tailed Student's *t*-tests. **P<0.01, ***P<0.001; n.s., not significant. (**D**) The let-7a/b activities were reconstituted by mimics. Relative mRNA abundance of MYC was examined by RT-qPCR assay, and c-Myc protein level tested by western blot. Error bars represented mean±s.d.

To investigate the molecular mechanism whereby p62 regulates the MYC mRNA level, we first examined whether p62 can directly regulate *MYC* transcription. To this end, we cloned the full sequence of *MYC* promoter (Figure 7A(a)) by PCR and inserted into luciferase reporter pGL3-basic plasmid through *Mlu* I and *Hind* III cloning sites. Dual-luciferase reporter results showed that luciferase firefly did not significantly change after p62 overexpression in both MDA-MB-231 (Figure 7A(b)) and MCF-7 cells (Supplementary Figure 7), suggesting that p62 does not influence the proximal *MYC* promoter activity.

We next asked whether p62 would affect MYC mRNA stability after transcription. We overexpressed p62 in MDA-MB-231 and MCF-7 cells, and then treated cells with Actinomycin D (ActD) to block *de novo* mRNA synthesis and total RNA was isolated at indicated time points (0, 30, 60 and 90 min) after ActD application. Relative MYC mRNA levels were measured by quantitative RT–PCR normalized to that at 0 min (using Actin as the internal control). As shown in Figure 7B, p62 was overexpressed in MDA-MB-231 cells, and that extended the half-life of MYC mRNA. *T*_1/2_ for the vector counterpart (Ctrl) and p62-WT in MDA-MB-231 cells was 38.68 and 70.88 min, respectively. Similar results were obtained in MCF-7 cells (Supplementary Figure 8). Taken together, these results suggest that p62 specially increases MYC mRNA stability after transcription, thus elevating its expression level.

Previous studies have reported that the let-7 cluster can bind to the consensus binding sites in the 3'untranslated region (UTR) of MYC mRNA transcript, leading to its degradation via the RNA miRNA-induced silencing complex.^41, 42, 43, 44, 45^ To test whether p62 regulates MYC expression via let-7 cluster, total RNA was extracted from MDA-MB-231 cells with or without transient p62 overexpression. RT-qPCR analysis of the let-7 cluster in the transfected cells showed that let-7a/b were significantly repressed by p62 overexpression (Figure 7C). Indeed, consistent with previous reports, transfection of let-7a/b mimics suppressed MYC expression in both MDA-MB-231 and MCF-7 cell lines (Supplementary Figure 9; Dicer protein level was detected to assess the efficiencies of the mimics). To determine whether the reduced expression of let-7a/b was responsible for p62-mediated stabilization of MYC mRNA, we co-transfected let-7a/b mimics with p62 in breast cancer cells. The results showed that re-constitution of let-7a/b activity by the mimics reversed the p62-mediated elevation of MYC at the mRNA and protein levels (Figures 7D(a) and (b)). Taken together, these results suggest that the microRNA let-7a/b have a critical role in the regulation of MYC mRNA stability and expression by p62.

## Discussion

In the present study, we uncover a previously unrecognized role of p62 in the regulation of breast cancer stem-like properties based on the following novel findings: (a) p62 expression is elevated in BCSC-enriched cell populations (Figure 1); (b) p62 is essential for promoting breast cancer stem-like properties both *in vivo* and *in vitro* (Figures 2 and 3, Supplementary Figure 5); (c) p62 is overexpressed in breast cancer tissues, and high p62 expression levels predict poorer clinical outcome (Figure 4); (d) p62 specially promotes MYC mRNA stability, mostly by repressing the expression of let-7a /b (Figures 5, 6, 7).

Given its contributions to tumor relapse and drug resistance, the BCSC population is considered as a key therapeutic target for breast cancer treatments. However, due to the heterogeneity and dynamic status of the BCSCs, there are hitherto no available strategies to eliminate BCSCs effectively. Thus, the elucidation of key molecules and mechanisms underlying BCSC regulation is urgently needed. We report here for the first time that BCSC-enriched populations (CD44^+^/CD24^−^ subpopulation, mammospheres, ALDH1^+^ populations and SP cells) display high levels of p62 expression (Figure 1). Further analysis confirmed that p62 is required for self-renewal and tumor-initiating abilities of BCSCs (Figures 2 and 3, Supplementary Figure 5). Notably, at least 1 × 10^5^ shp62 MDA-MB-231 cells were required to get 33% tumor graft rate (1/3 sites), whereas as few as 1 × 10^3^ shNC MDA-MB-231 cells could robustly initiate tumors (2/3 sites) within the same time frame. Previous reports point out that p62 is partially involved within cisplatin resistance in human ovarian cancer cells, possibly by modulating Keap1-NRF2-ARE signaling activities.^46^ Indeed, our results also revealed that suppression of p62 reduced the mammosphere-forming ability of the epirubicin-resistant MCF-7 cells (Figure 6D), a cell line that shows aggressive stem-like features and multidrug resistance.^21^ Collectively, our data strongly suggest p62 as a potential target to limit breast cancer stemness, thus overcoming BCSC-mediated drug resistance.

p62 has been recently reported to exert a role in controlling mitosis transition through CDK1-mediated phosphorylation at Threonine 269 (T269) and Serine 272 (S272).^47^ p62 can bind to and inhibit Twist-1 autophagic degradation, thus promoting xenografted human skin cancer cell growth in a Twist-dependent manner.^48^ In our study, we performed BrdU, cell-cycle and apoptosis assays to investigate whether the effects of p62 arisen from its influence on cell short-term proliferation and viability. The results showed that there were no significant differences in the number of BrdU-positive cells in both the p62 knockdown MDA-MB-231 cells and the p62 overexpression MCF-7 cells when compared with their control counterparts (Supplementary Figure 11A(a) and (b)). In addition, cell-cycle assays indicated that there were no changes in G0/G1, S and G2/M phase cells following the depletion or overexpression of p62 (Supplementary Figure 11B(a) and (b)). Together with the results shown in Supplementary Figure 10, which indicate that neither forced expression nor depletion of p62 impaired the growth rate of breast cancer cells in a short time frame, as indicated by proliferative assays of up to 4th–6th day, suggesting that p62 is not capable of influencing the short-term proliferative ability of breast cancer cells. Consistent with our findings, one recent study has shown that cell growth rates are not altered till the 4th day by shRNA-mediated suppression of p62 in mouse embryo fibroblasts.^49^ Moreover, apoptosis detection by Annexin-V/PI using flow cytometry showed no changes following p62 depletion or overexpression in breast cancer cells (Supplementary Figure 12). Therefore, these findings led us to conclude that the reduced spheroid formation and tumor-initiating abilities caused by p62 knockdown were independent of the function of p62 to induce cell proliferation and survival in breast cancer. In longer term experiments, suppression of p62 decreased colony-forming abilities (Figure 2B and Supplementary Figure 1C), as well as reduced Ki67-positive rate in tumor xenografts (Supplementary Figure 4). One possible explanation for the apparent contradiction might be the diverse culture conditions. Plate colony formation reflected the ability of one single cell to self-renewal over long periods of time (~14 days), while tumor cell transplantation studies reveal stem-like properties (at least 28 days).

Recent studies suggest that autophagy has a crucial role in the origin, maintenance and systemic distribution of BCSCs.^50^ Here, we examined the markers of autophagy following p62 depletion or overexpression in breast cancer cells. Surprisingly, LC3 conversion was not significantly changed by either knockdown or overexpression of p62, indicating that p62 does not alter autophagic activities under normal culture conditions (Supplementary Figure 13A). In addition, we labeled the single cells, which were isolated from the third generation of spheroids from shNC and shp62 groups, with an autophagic vacuole dye, monodansylcadaverine. As shown in Supplementary Figure 13B, there were no significant differences in the number of autophagic vacuoles formed in shNC and shp62 cells, indicating that p62-mediated mammosphere-forming capacity is not primarily regulated by autophagy. To further assess whether autophagy has a role in regulating the p62-mediated stem-like properties in breast cancer, we conducted shBeclin1-mediated knockdown of autophagy in both MDA-MB-231-shNC and shp62 cells. Inhibition of autophagy resulted in a significant increase in the mammosphere-forming capacity in MDA-MB-231-shNC cells (Supplementary Figure 14). Critically, suppression of autophagy in MDA-MB-231-shp62 cells could not reverse the reduction in mammosphere-forming ability induced by p62 depletion (Supplementary Figure 14), suggesting that autophagy is unlikely to have a key role in mediating the p62-induced stem-like properties in breast cancer. However, we cannot absolutely rule out the possibility that autophagy is not involved in modulating the p62-mediated breast cancer stem-like properties, due to the dynamic nature of BCSCs and microenvironmental stresses.^50^

In an effort to investigate the mechanisms underlying the regulation of MYC expression by p62, we found that p62 enhances MYC mRNA expression at the post-transcriptional level rather than through promoting its transcriptional activity (Figure 7). MYC mRNA stabilization is mainly determined by two intrinsic instability elements, localizing in the 5' and 3' UTR, respectively. The 5'UTR element is composed of 249 nucleotides, also termed as coding region determinant, which can be recognized by endonucleases, thus promoting MYC mRNA degradation. Several proteins have been assigned to stabilize MYC mRNA by specifically recognizing and binding to the CRD, thus preventing endonucleolytic cleavage of MYC mRNA, such as the Coding Region Determinant-Binding Protein,^51, 52, 53^ and IGF2BP1.^54^ The 3'UTR instability element is rich in adenine and uracil (AURE, AU-rich element). Both HUR and let-7 have been reported to bind to the 3'-element, thus destabilizing MYC mRNA level and repressing its expression.^55, 56^ In our study, we found that forced p62 expression significantly delayed half-life of MYC mRNA in breast cancer cell lines (Figure 7B and Supplementary Figure 8), indicating a role of p62 in stabilizing MYC mRNA. However, we failed to detect a direct binding of p62 with MYC mRNA (Supplementary Figure 15), ruling out the possibility that p62 acted as an RNA-binding protein to stabilize MYC mRNA directly. Intriguingly, we uncover here that let-7a and let-7b act as a key effector in the regulation of MYC mRNA stability by p62 (Figures 7C and D). Let-7 is a conserved microRNA family, which can function as a tumor suppressor.^44, 45, 57^ Our data suggested a negative feedback correlation between p62 and the let-7 microRNA family (Figure 7C), further confirming the oncogenic role of p62 in breast cancer. Nevertheless, the exact mechanism accounting for the p62-mediated repression of let-7a and let-7b requires further evaluation. Meanwhile, given that p62 can activate mTORC1 signaling in response to amino-acid stimuli,^26^ and c-Myc has been found to be a key transcription factor regulated by mTORC1 signaling,^58^ we also tested the role of mTORC1 signaling in p62-mediated stabilization of MYC mRNA. Although rapamycin (a small-molecule inhibitor of mTORC1 kinase) effectively inhibited MYC expression (Supplementary Figure 16a), it did not block the elevation of c-Myc protein by p62 overexpression (Supplementary Figure 16b), suggesting that mTORC1 signaling is not likely to be involved in the regulation of MYC mRNA stability by p62 in our system.

In summary, we demonstrate that the signaling adaptor p62-promoted breast cancer stem-like properties through stabilizing MYC mRNA at the post-transcriptional level. Our findings also suggest that target p62 might be a promising avenue for therapeutic intervention for overcoming BCSC-mediated drug resistance and tumor relapse.

## Materials and methods

### Cell culture

The human breast cancer cell lines (MDA-MB-231, BT-549, MCF-7 and SKBR-3), the immortalized human breast epithelial cell line MCF-10A and human embryonic kidney HEK293T cell line were obtained from the American Type Culture Collection (ATCC). The epirubicin-resistant MCF-7 cells (EpiR-MCF-7) have previously been described.^59^ The SUM149 cell line was kindly provided by Prof. Zhi-Min Shao (Department of Medical Oncology, Cancer Hospital of Fudan University, Shanghai Medical College, Shanghai, China). Cell lines were acquired from the Cell Culture Service, Cancer Research UK, where it was tested and authenticated, and were not cultured continuously for more than 3 months. Each cell line was cultured in its standard medium as recommended by ATCC. SUM149 cells were cultured in F-12 Hams (Gibco, New York, NY, USA) supplemented with 5% FBS (Hyclone, Logan, UT, USA), 5 μg/ml insulin (Sigma-Aldrich, St Louis, MO, USA) and 1 μg/ml hydrocortisone.

### Patients and follow-up, tissue microarray and IHC assay

This study comprises 369 female breast cancer patients diagnosed between August 1999 and October 2008 at Sun Yat-sen University Cancer Center. All patients were staged according to the American Joint Committee on Cancer (AJCC) TNM staging system for breast cancer (seventh edition). Tissue microarray sections were constructed, and immunohistochemical analysis was performed as previously described.^60^ Detailed information is described in Supplementary Materials and Methods.

### RNAi and microRNA transfection

Transient RNAi transfection was carried out as previously described.^61^ Two different target siRNA sequences of p62 were obtained from GenePharma Co., Ltd (Shanghai, China) (1#, 5′-GUGACGAGGAAUUGACAAUTT; 2#, 5′-GGAGUCGGAUAACUGUUCATT). The negative control siRNA was 5′-UUCUCCGAACGUGUCACGUTT. The let-7a mimic (5′-UGAGGUAGUAGGUUGUAUAGUU; 5′-CUAUACAACCUACUACCUCAUU) and the let-7a mimic (5′-UGAGGUAGUAGGUUGUGUGGUU; 5′-CCACACAACCUACUACCUCAUU) were obtained from Invitrogen and transfected into the culture cells using Lipofectamine2000 Transfection Reagent (Invitrogen, Carlsbad, CA, USA) according to the manufacturer's instructions.

### Plasmid construction

Full length of p62 fragment was cloned from human genome cDNA and ligated into pCDH vector (System Biosciences, Palo Alto, CA, USA). The primers were as follows: p62: 5' *Xba*I, 5′-GCTCTAGAGCGCCACCATGGCGTCGCTCACCGTGAAGGCC; 3′ *Eco*RI, 5′-CCGGAATTCCGTCACAACGGCGGGGGATGCTTTGA. The *MYC* promoter fragment was cloned from human genomic DNA and ligated into the pGL3-basic vector (Promega, Madison, WI, USA) upstream of the luciferase reporter. Primers were as follows: 5′ *Mlu*I, 5′-CGACGCGTCGGCCACCGGGAGAGAAAAGTTTACT; 3′ *Hin*dIII, 5′-CCCAAGCTTGGGCTGGTTTTCCACTACCCG. The full-length MYC plasmid was kindly provided by Dr Fei-Meng Zheng (State Key Laboratory of Oncology in South China, Cancer Center, Sun Yat-sen University, Guangzhou, China) and packaged for lentivirus particles. The shRNA targeting p62 (1#, 2#) and the non-target shRNA (SHC002) were kindly provided Dr Zi-Jie Long (Department of Hematology, the Third Affiliated Hospital, Sun Yat-sen University, Guangzhou, China) and packaged for lentivirus particles. Lentiviral particles containing shBeclin1 (131209AZ) and LV3 (131127CZ) control expression vectors were purchased from Shanghai GenePharma Co., Ltd. For lentivirus preparation, see Supplementary Materials and Methods.

### Gene expression profiling analysis and Bioinformatics analysis

Total RNA was extracted from both shNC and shp62 groups of xenografted tumor tissues using Trizol reagent and subjected to AFFYMETRIX Human Primeview array (Gene Tech (Shanghai) Company Limited) to determine the global expression of mRNA. Each group was tested in triplicate. GSEAs were performed using GSEA v2.0.13 software (http://www.broad.mit.edu/gsea).^62^ The gene expression signatures were acquired from the GSEA website (V$MYC_Q2, V$MYCMAX_B and V$MYCMAX_01). Statistical significance was evaluated by means of false discovery rate (⩽0.25) and *P*-value calculations (*P*<0.05).

For Mammosphere formation assay, SP assay, Cell plate colony formation assay, Animal studies, RNA extraction, Reverse transcription-PCR, Real-time quantitative PCR, Dual-luciferase reporter assay, Western blot analysis, RNA and protein immunoprecipitation and Statistical analysis, see Supplementary Materials and Methods.

## Figures and Tables

**Table 1:** Association of p62 expression with clinicopathological characteristics in breast cancer patients (*n*=369)

*Variable*	*All cases, N (%)*	*p62 expression*		*P*^a^
		Low	High	
*Age (years)*				0.614
<48	173 (46.9)	89	84	
⩾48^b^	196 (53.1)	106	90	
*Clinical stage*				0.011
I	76 (20.6)	46	30	
II	173 (46.9)	96	77	
III	117 (31.7)	52	65	
IV	2 (0.5)	0	2	
Missing cases	1 (0.3)			
*T stage*				0.700
T_1_	126 (34.1)	69	57	
T_2_	192 (52.0)	101	91	
T_3_	25 (6.8)	10	15	
T_4_	25 (6.8)	14	10	
Missing cases	1 (0.3)			
*Node stage*				0.004
N_0_	167 (45.3)	102	65	
N_1_	98 (26.6)	49	49	
N_2_	71 (19.2)	28	43	
N_3_	32 (8.6)	15	17	
Missing cases	1 (0.3)			

a *x*^2^ test.

b Median age.

**Table 2:** Results of multivariate Cox proportional-hazards analysis for overall survival (OS) and disease-free survival (DFS) in breast cancer patients (*n*=369)

*Variable*	*For OS*			*For DFS*		
	*Hazard ratio*	*95% Confidence interval*	P	*Hazard ratio*	*95% Confidence interval*	P
Age <48.00 (vs ⩾48 years)	0.554	0.236–1.301	0.175	0.782	0.382–1.598	0.500
Tumor stage T_3_+T_4_ (vs T_1_+T_2_)	1.413	0.667–2.995	0.367	1.037	0.500–2.148	0.923
Node stage N_2_+N_3_ (vs N_0_+N_1_)	1.927	0.846–4.387	0.367	3.435	1.555–7.588	0.002
Clinical stage III+IV (vs I+II)	2.391	1.123–5.091	0.024	1.762	0.928–3.348	0.083
p62 High (vs Low)	4.669	2.313–9.426	0.000	2.040	1.156–3.602	0.014
