# Supplementary figures and images for: p62/SQSTM1 enhances breast cancer stem-like properties by stabilizing MYC mRNA
Source: Oncogene. 2016 Jun 27;36(3):304–17. doi: 10.1038/onc.2016.202 (PMC5269535; doi:10.1038/onc.2016.202)

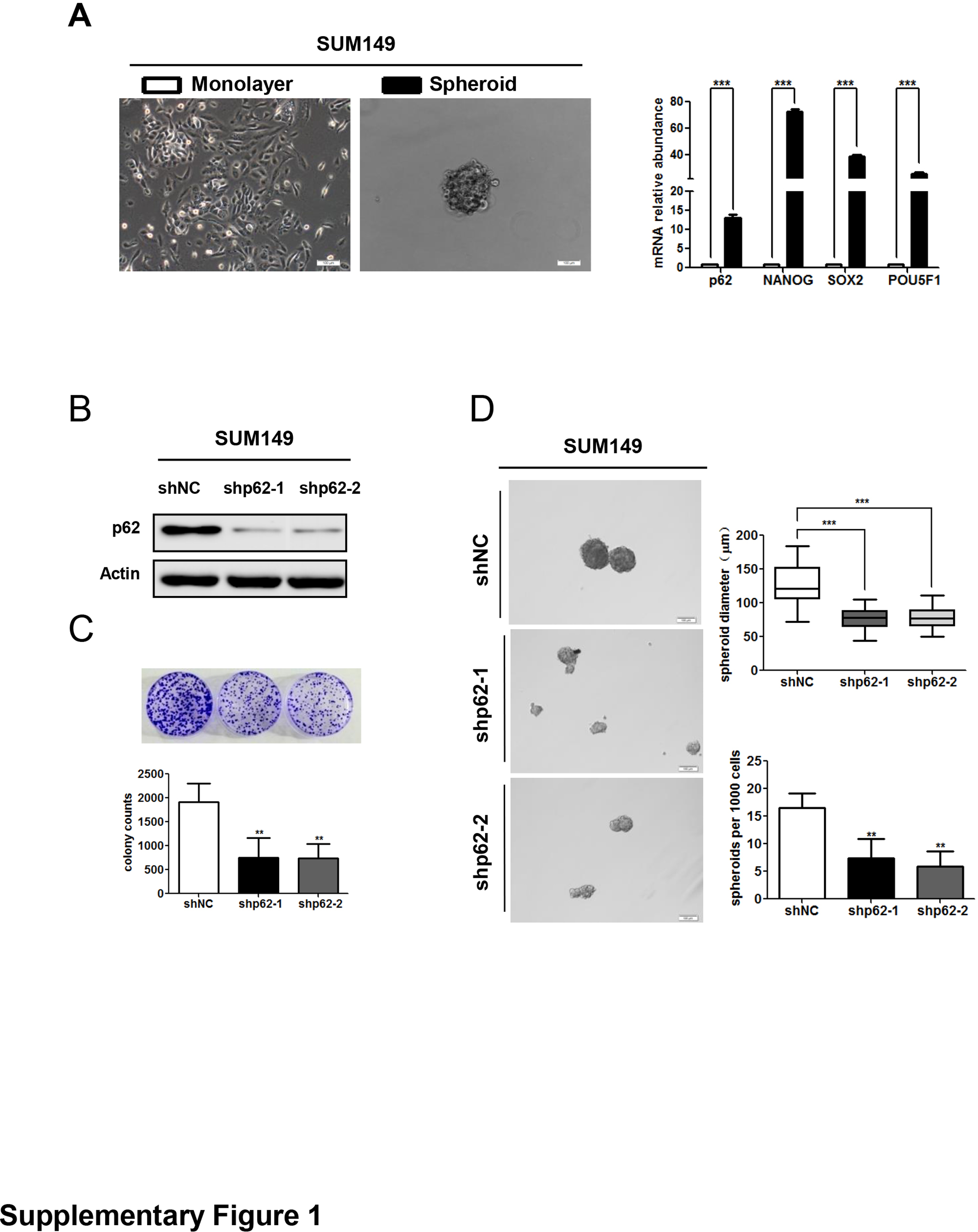

Supplement: Supplementary Figure 1 [file onc2016202x1.tif]

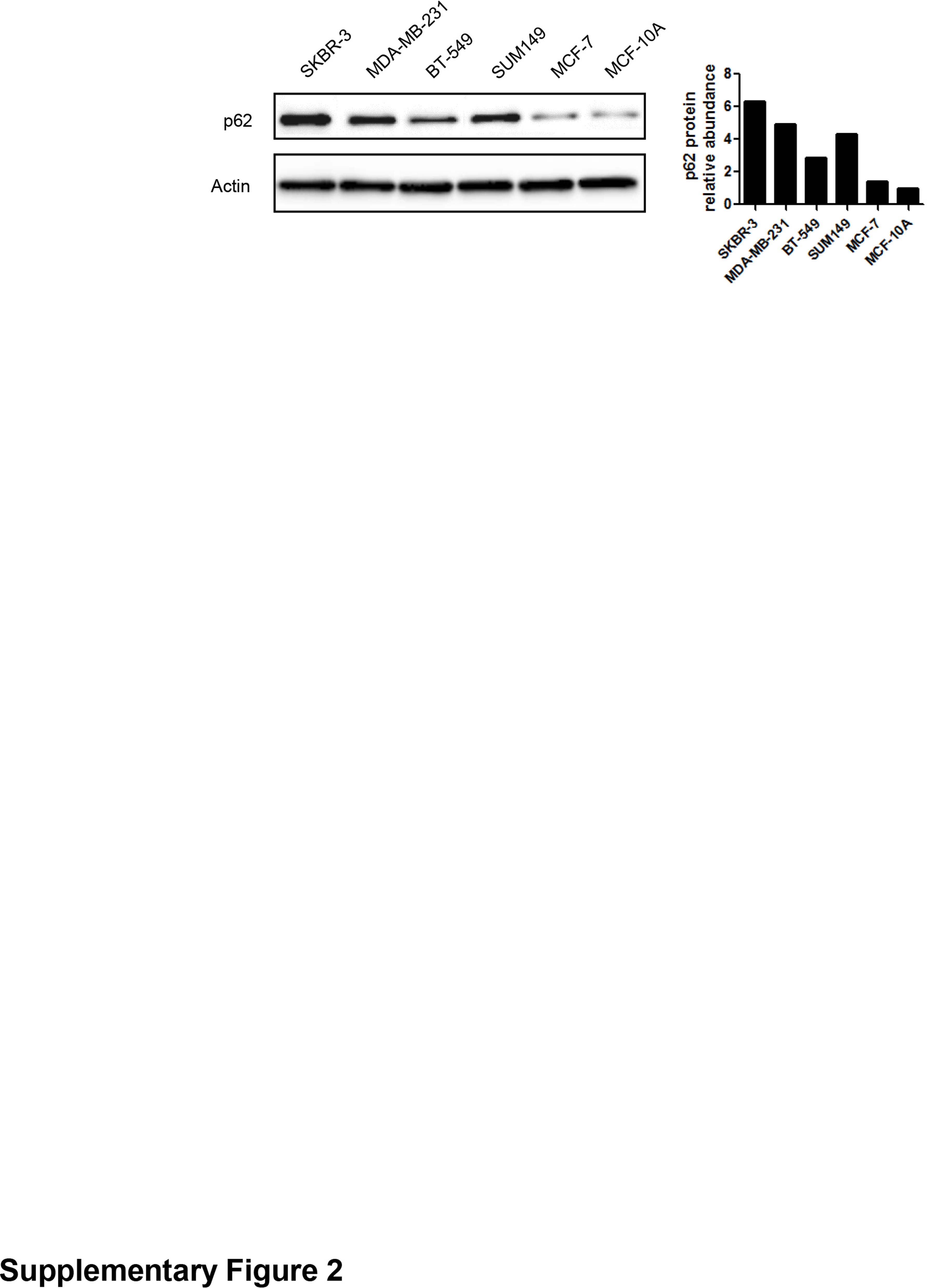

Supplement: Supplementary Figure 2 [file onc2016202x2.tif]

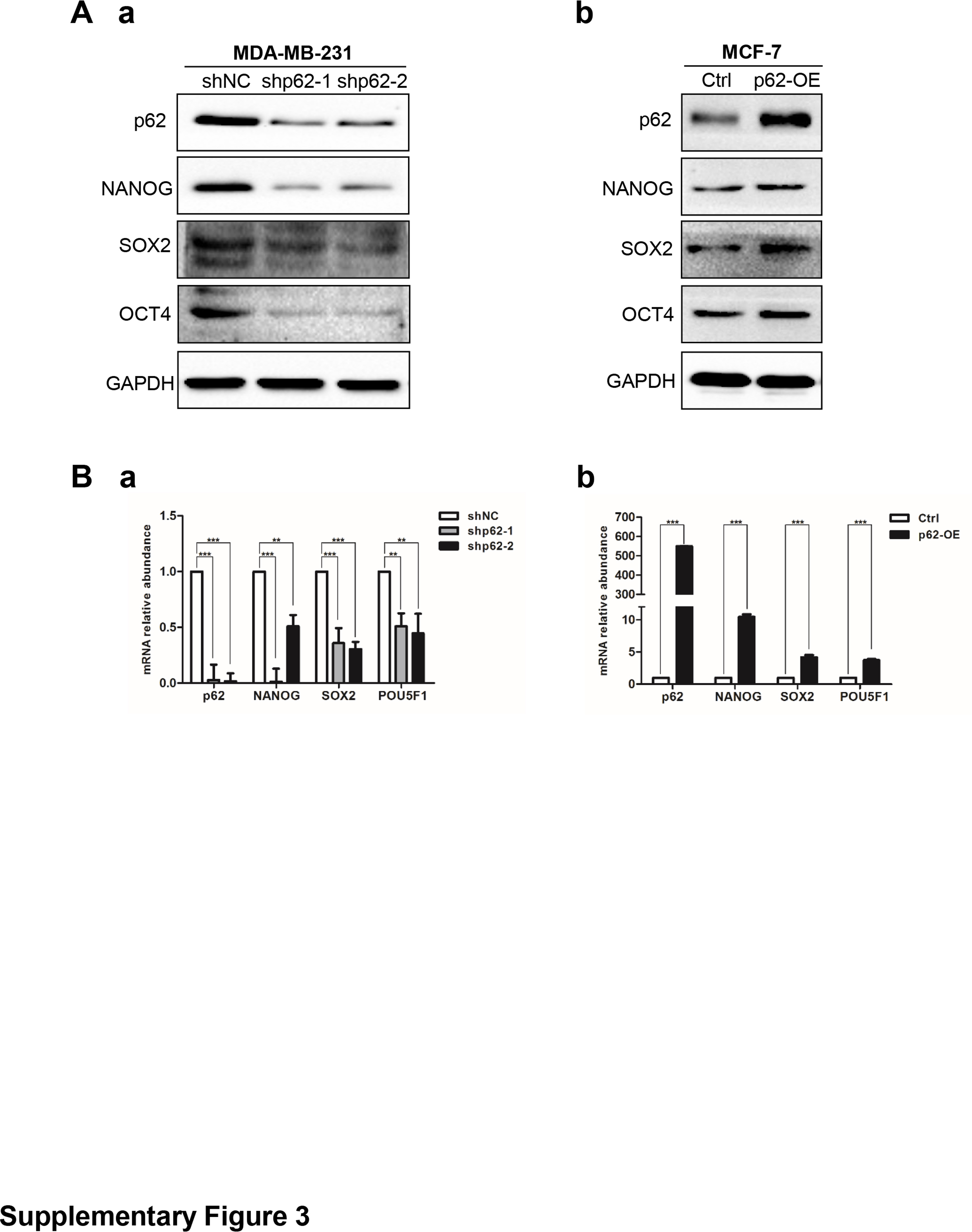

Supplement: Supplementary Figure 3 [file onc2016202x3.tif]

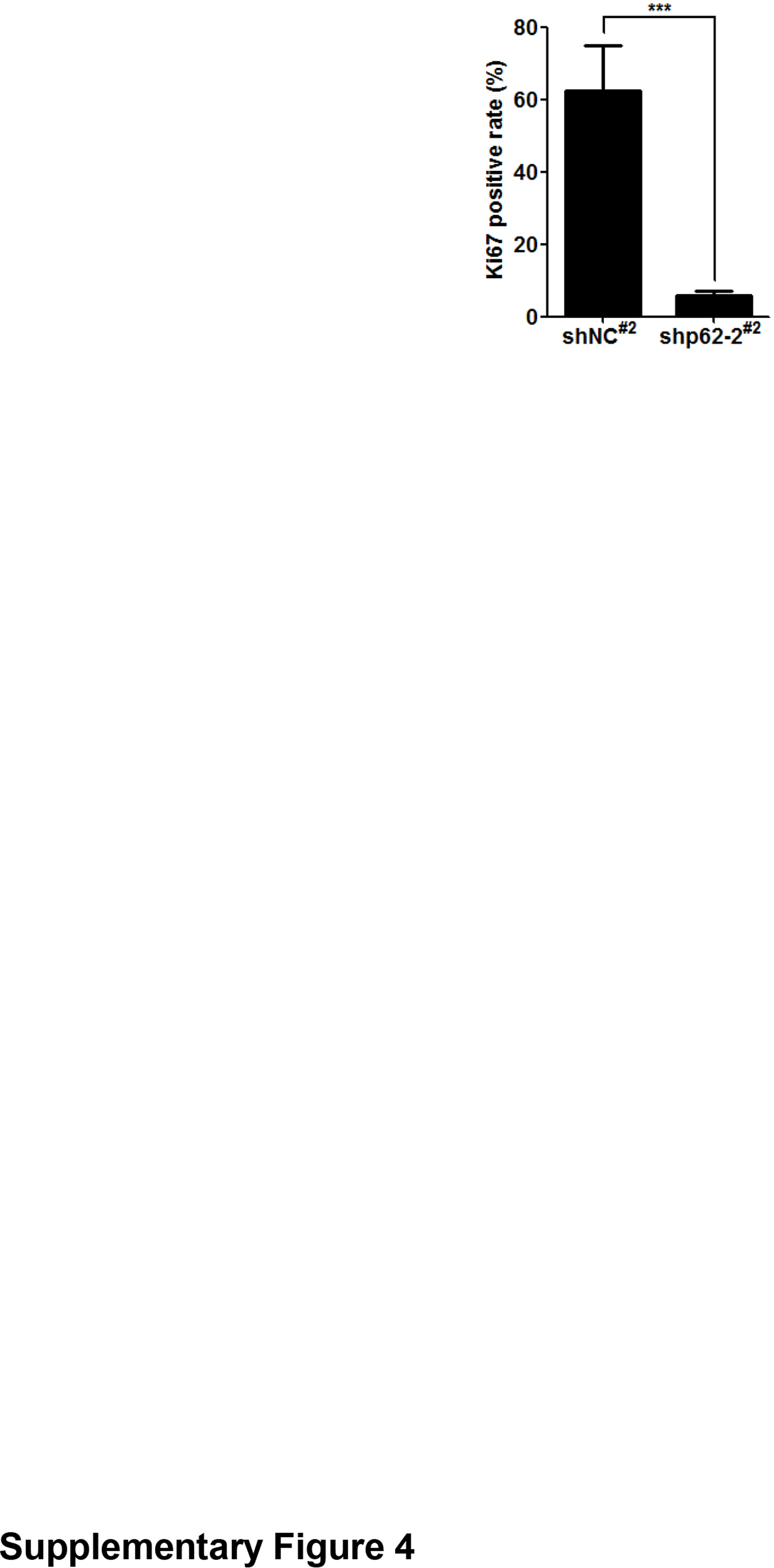

Supplement: Supplementary Figure 4 [file onc2016202x4.tif]

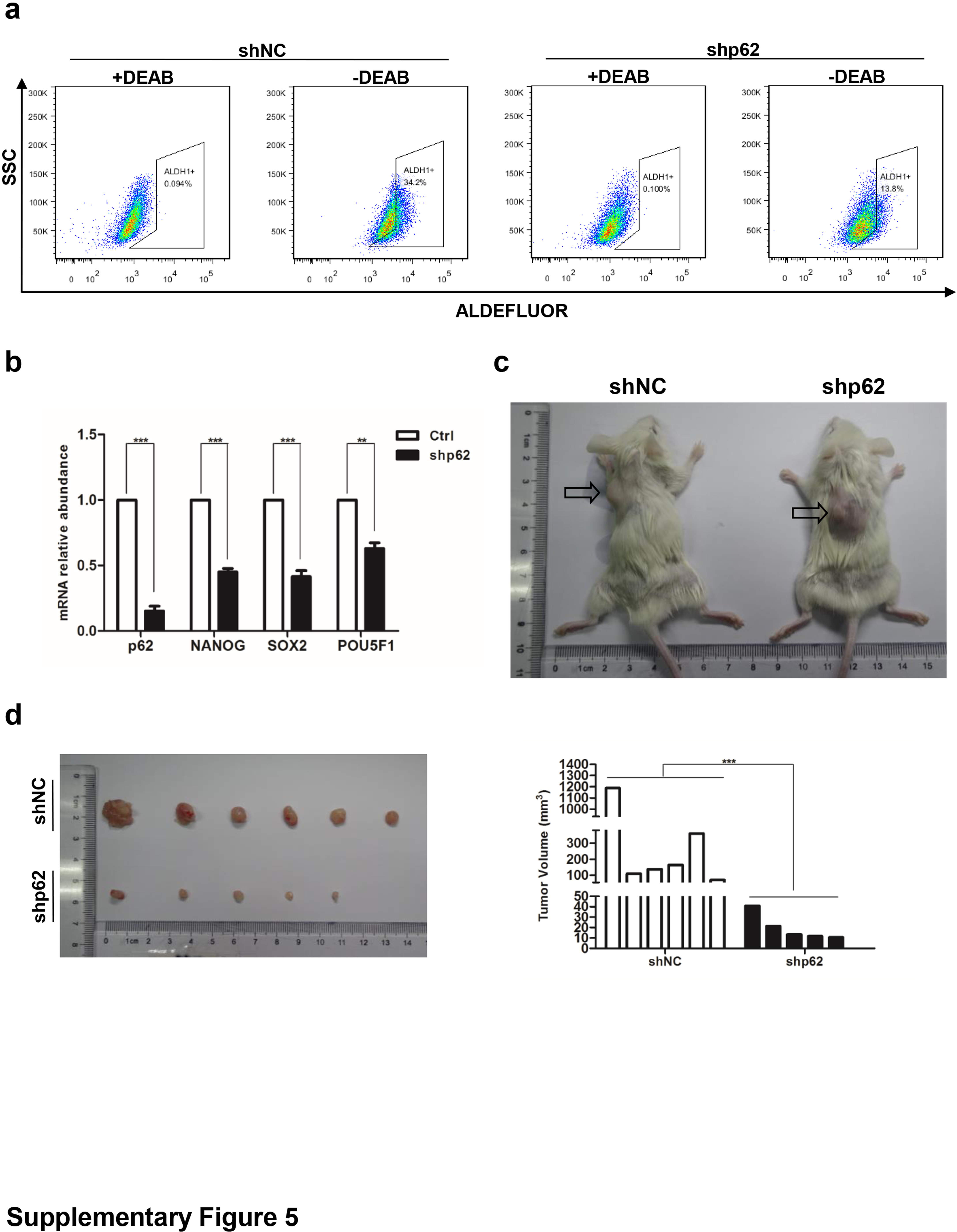

Supplement: Supplementary Figure 5 [file onc2016202x5.tif]

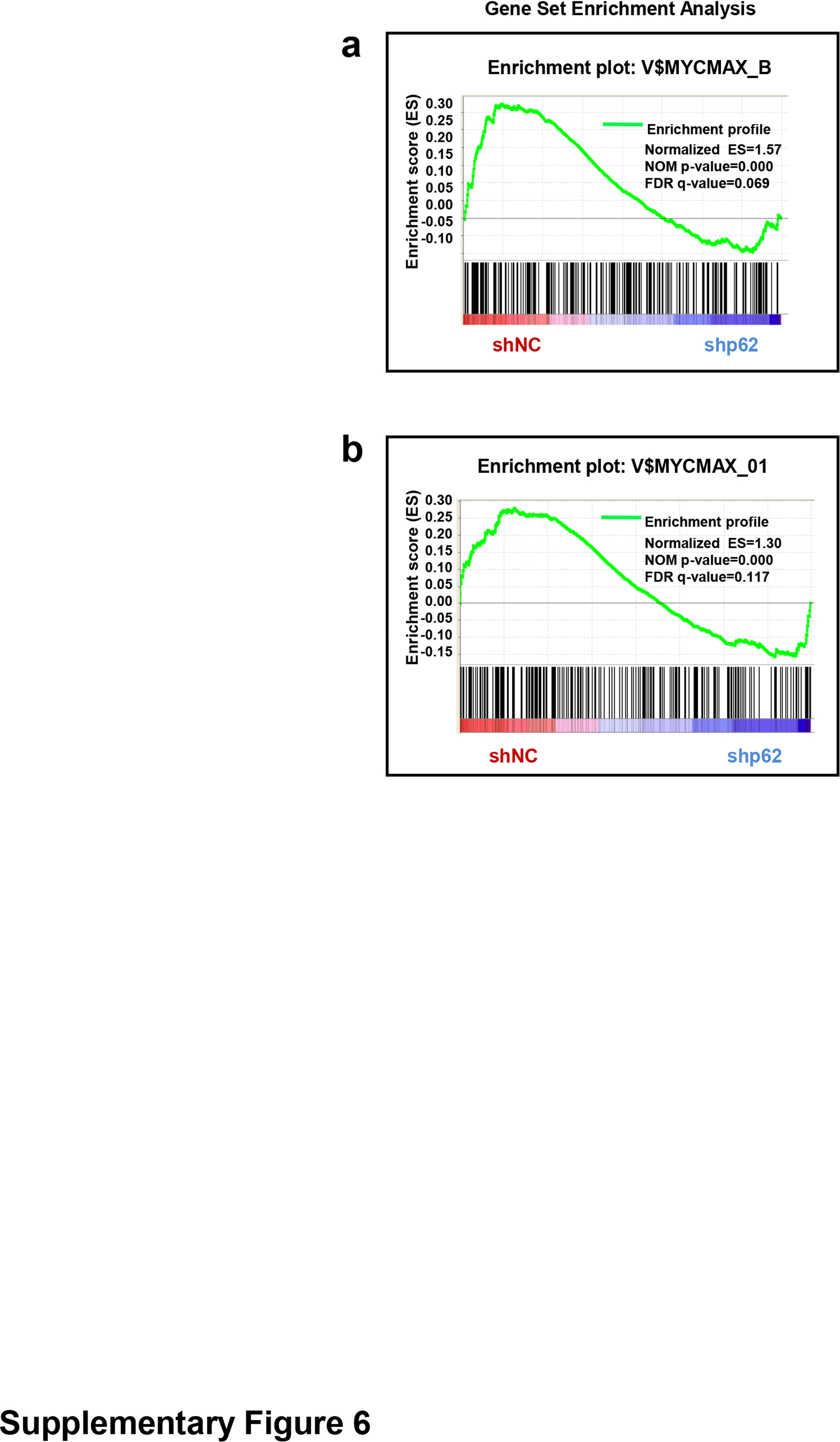

Supplement: Supplementary Figure 6 [file onc2016202x6.tif]

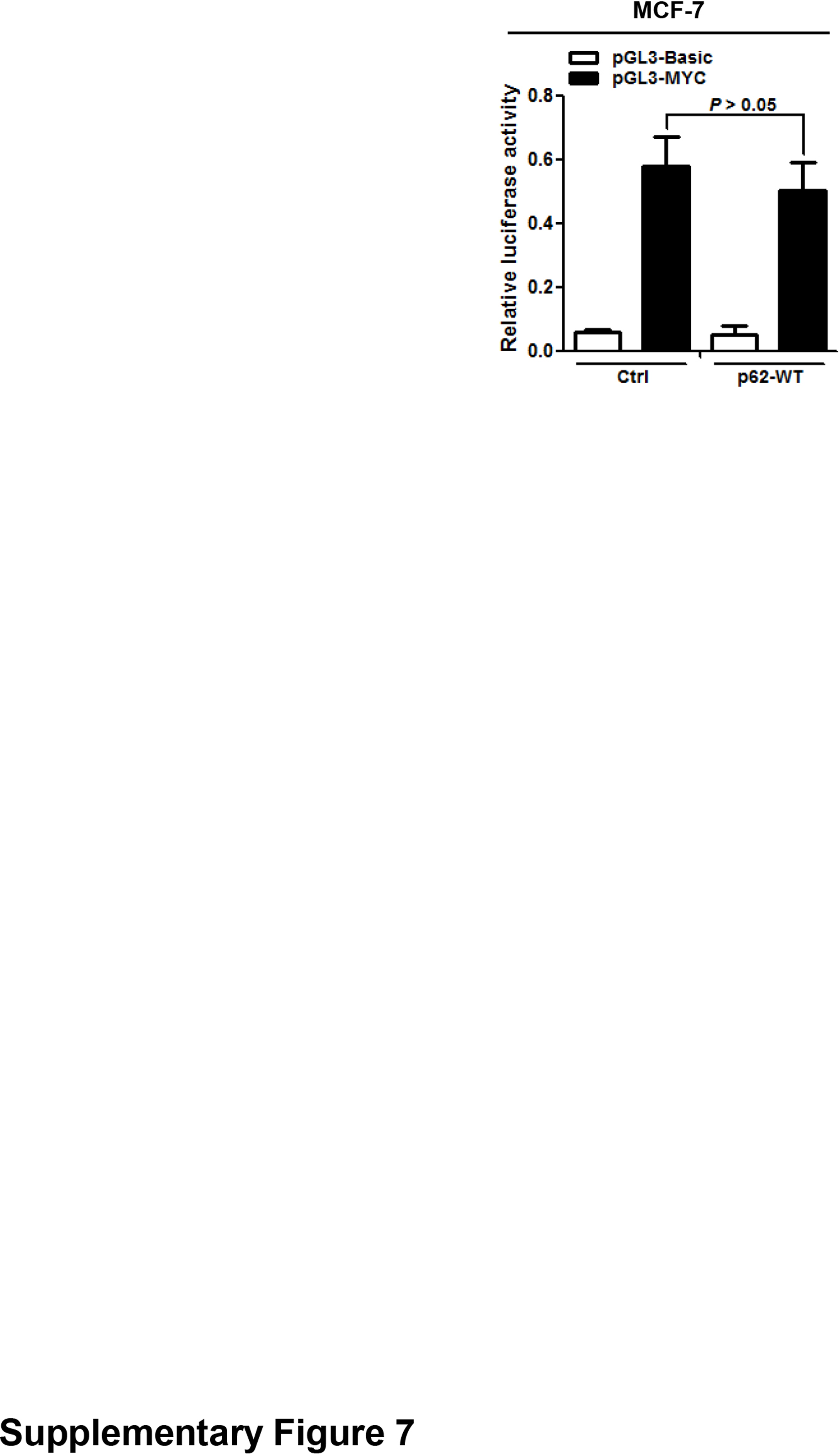

Supplement: Supplementary Figure 7 [file onc2016202x7.tif]

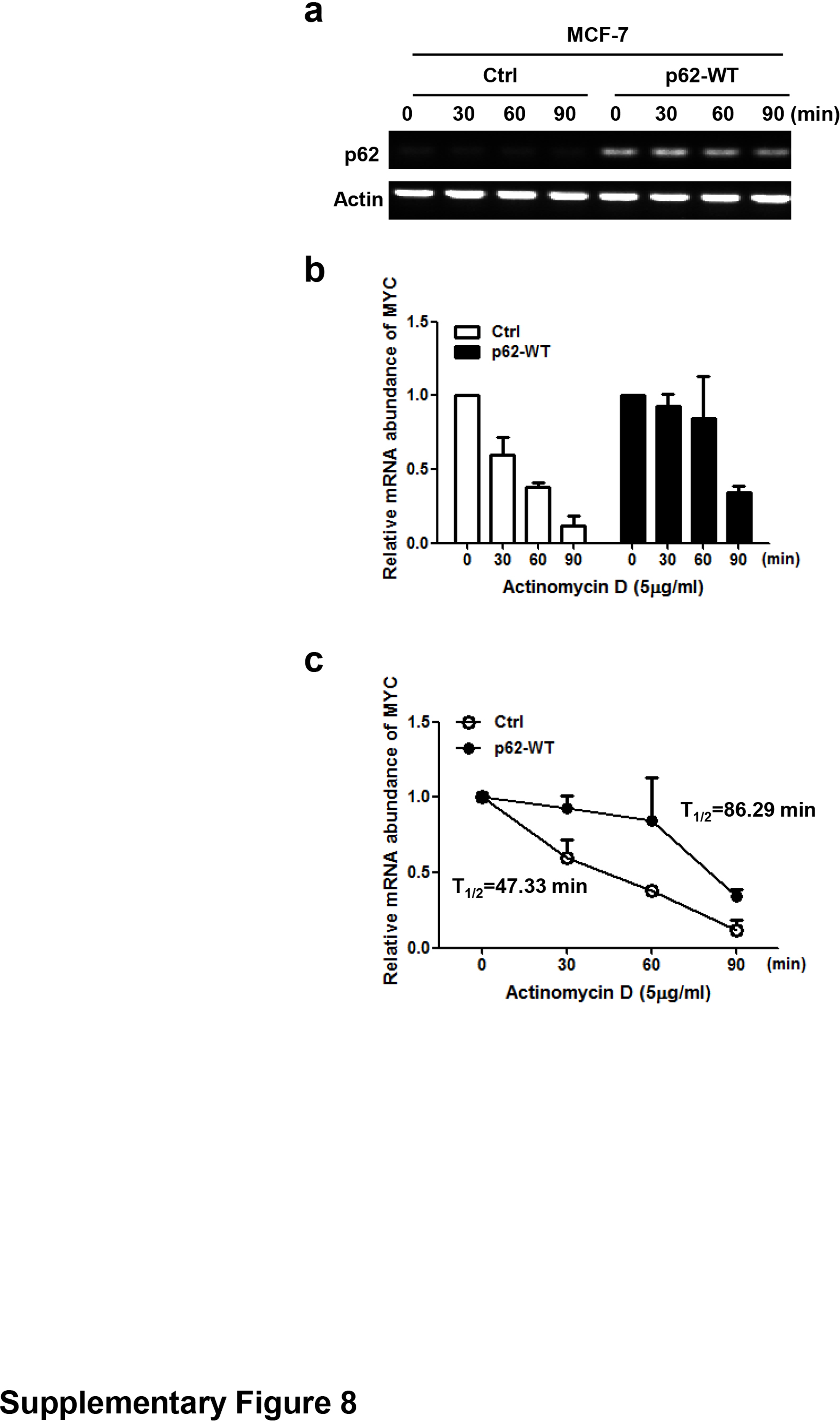

Supplement: Supplementary Figure 8 [file onc2016202x8.tif]

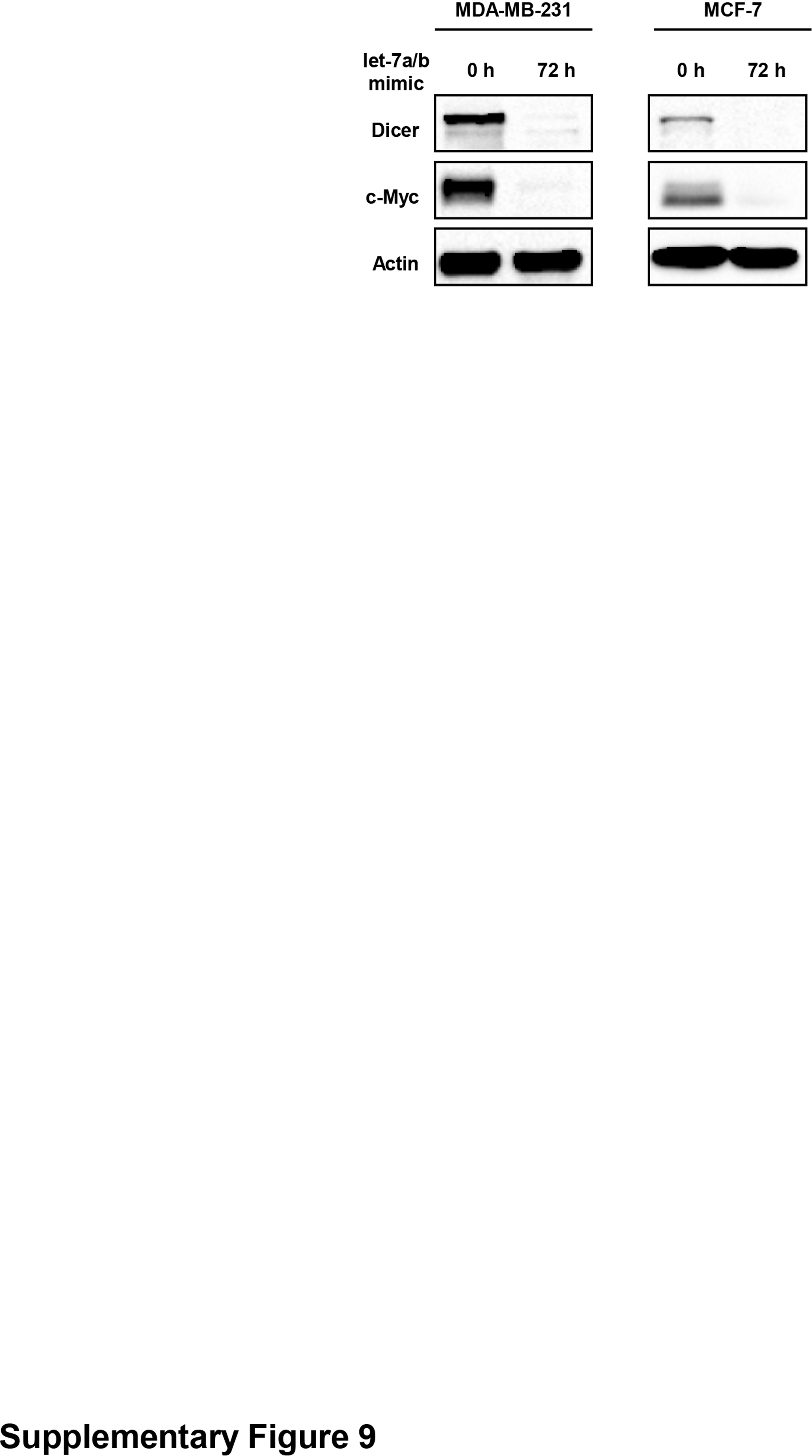

Supplement: Supplementary Figure 9 [file onc2016202x9.tif]

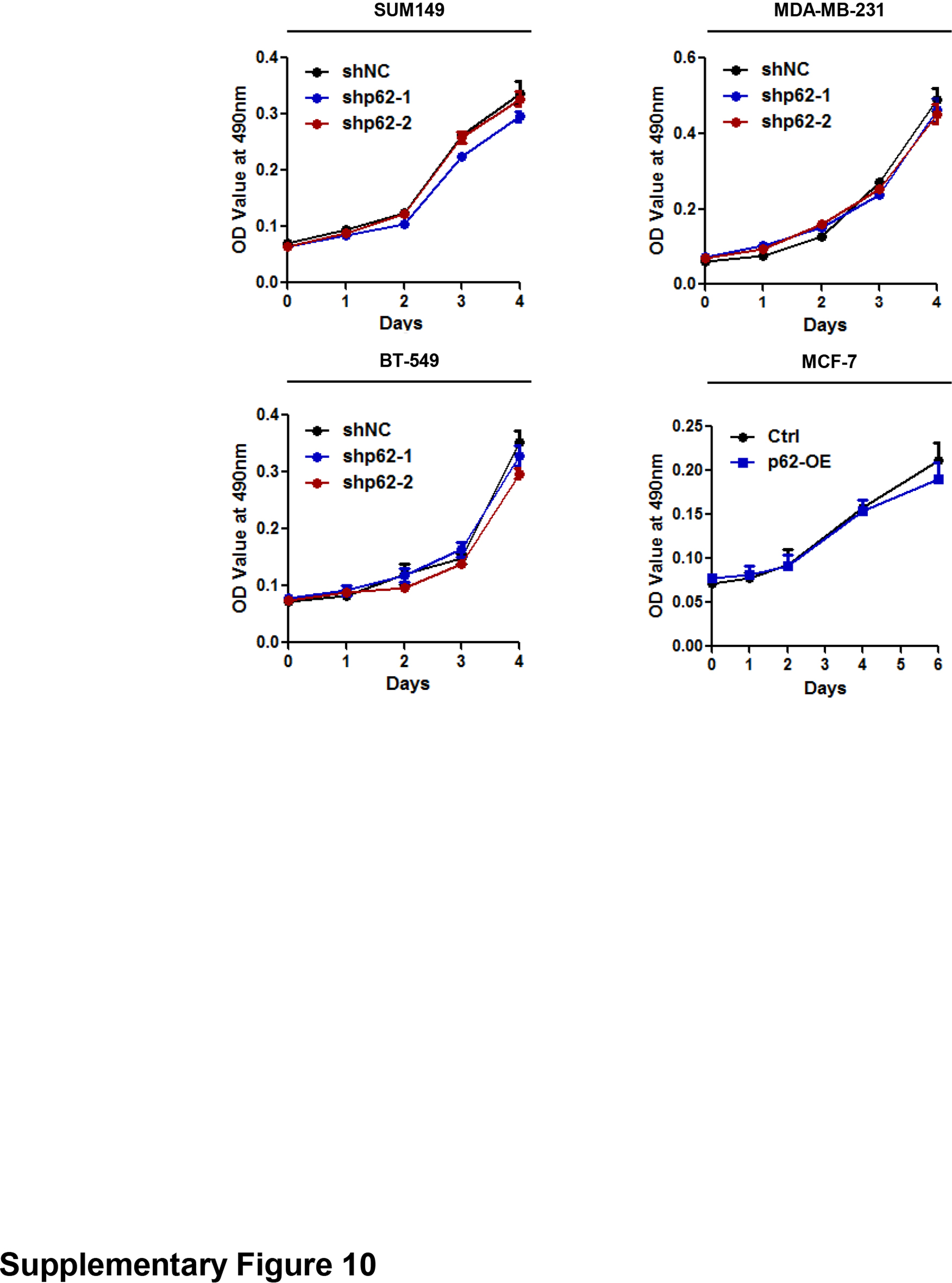

Supplement: Supplementary Figure 10 [file onc2016202x10.tif]

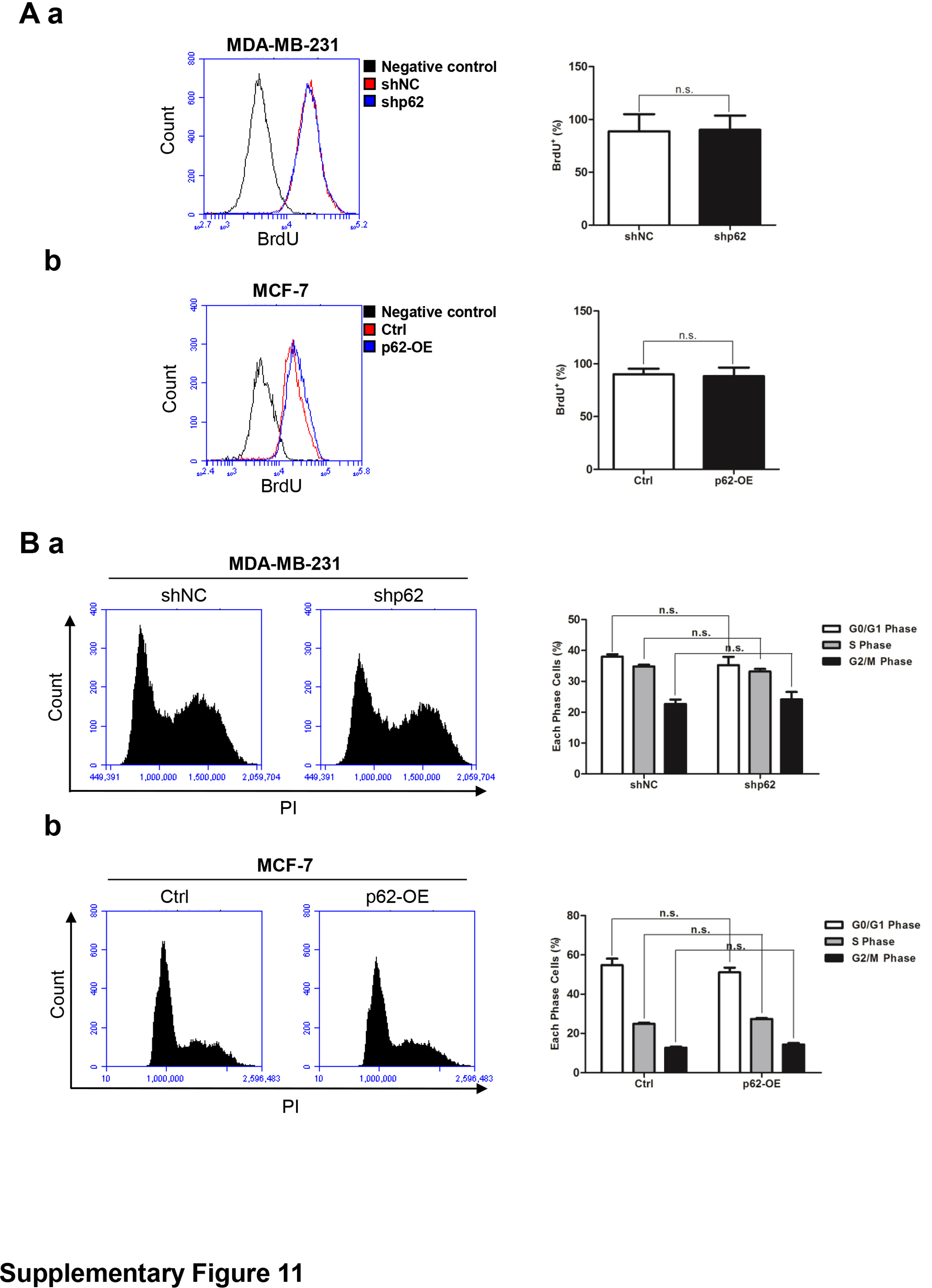

Supplement: Supplementary Figure 11 [file onc2016202x11.tif]

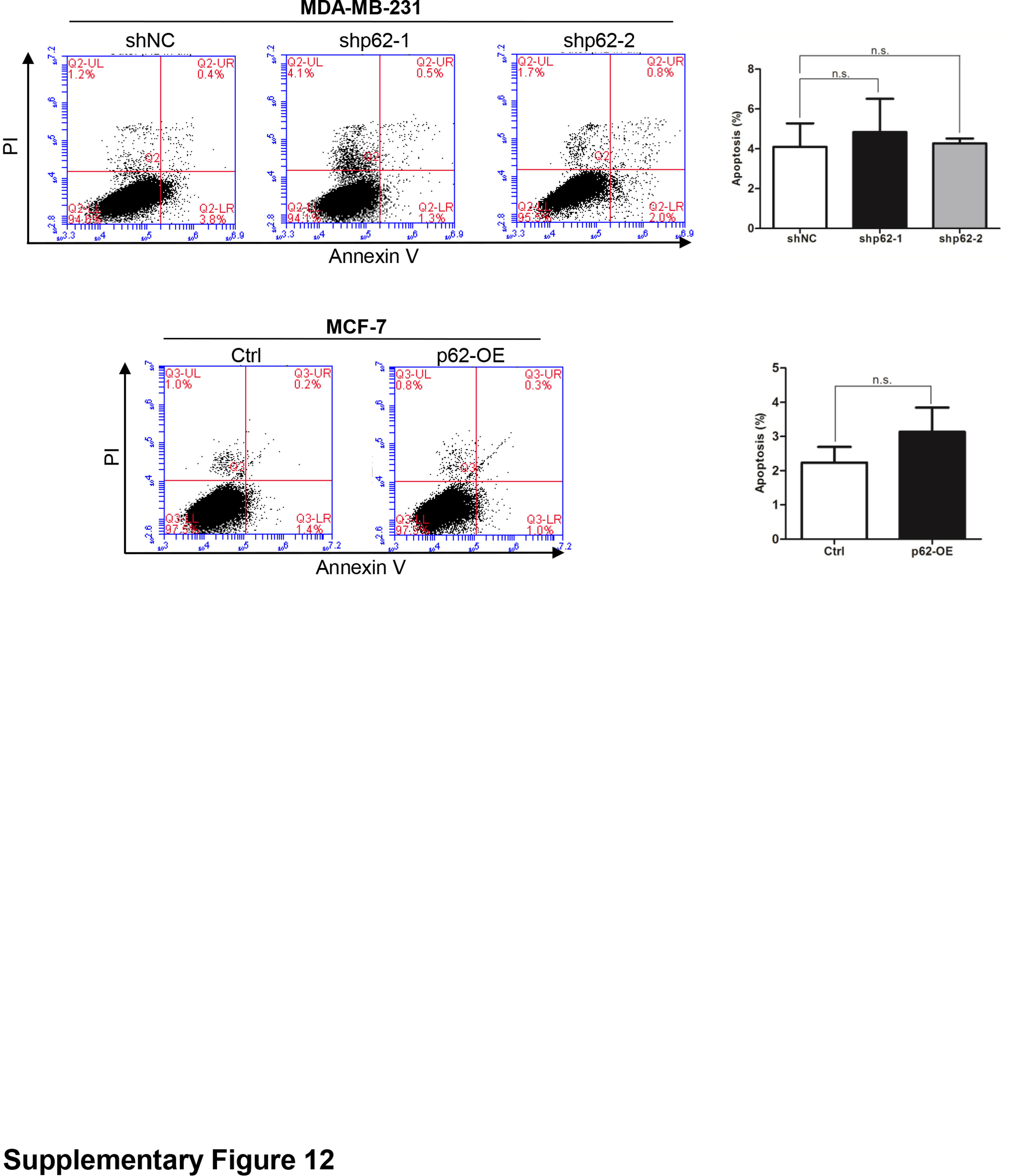

Supplement: Supplementary Figure 12 [file onc2016202x12.tif]

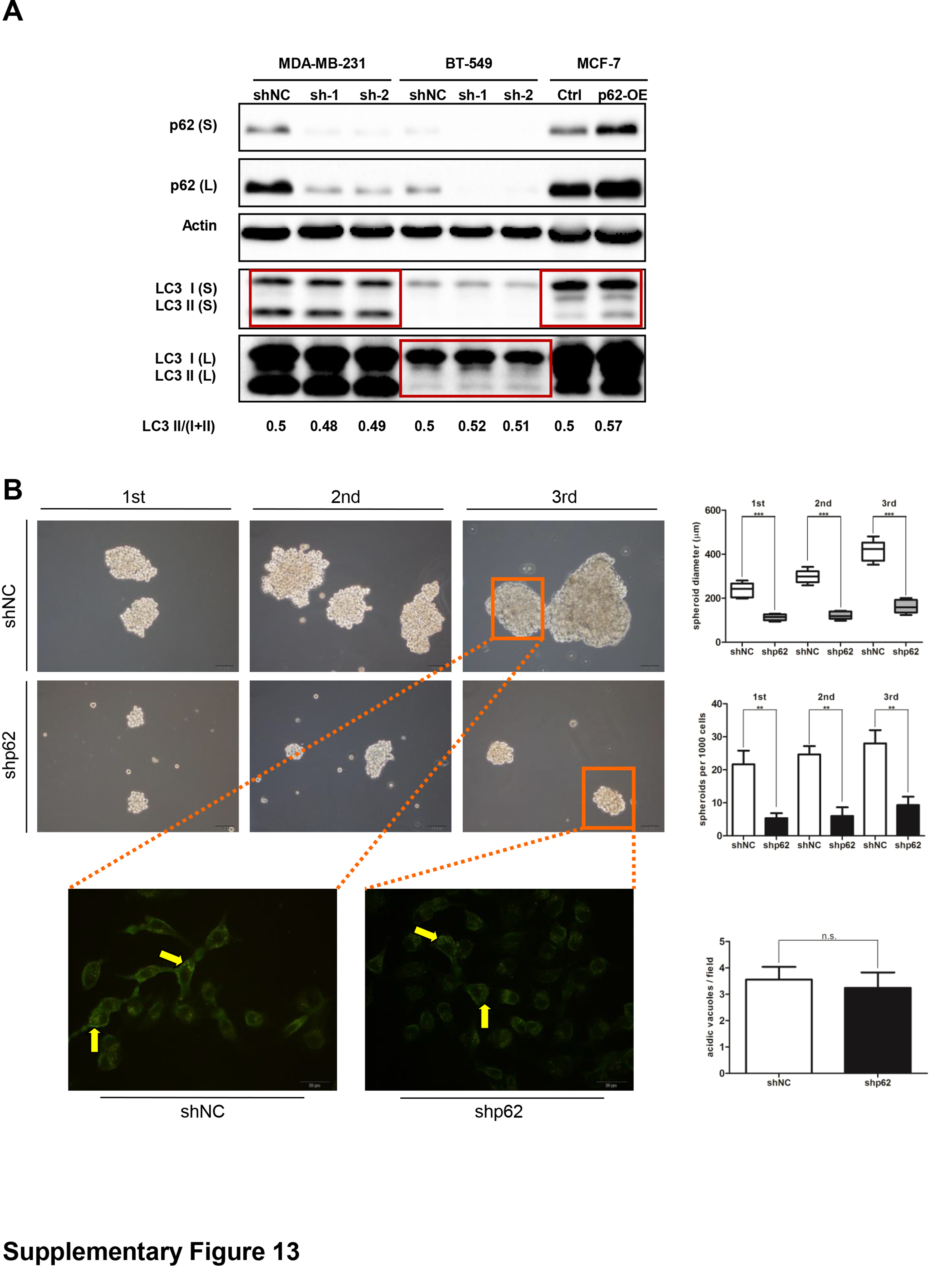

Supplement: Supplementary Figure 13 [file onc2016202x13.tif]

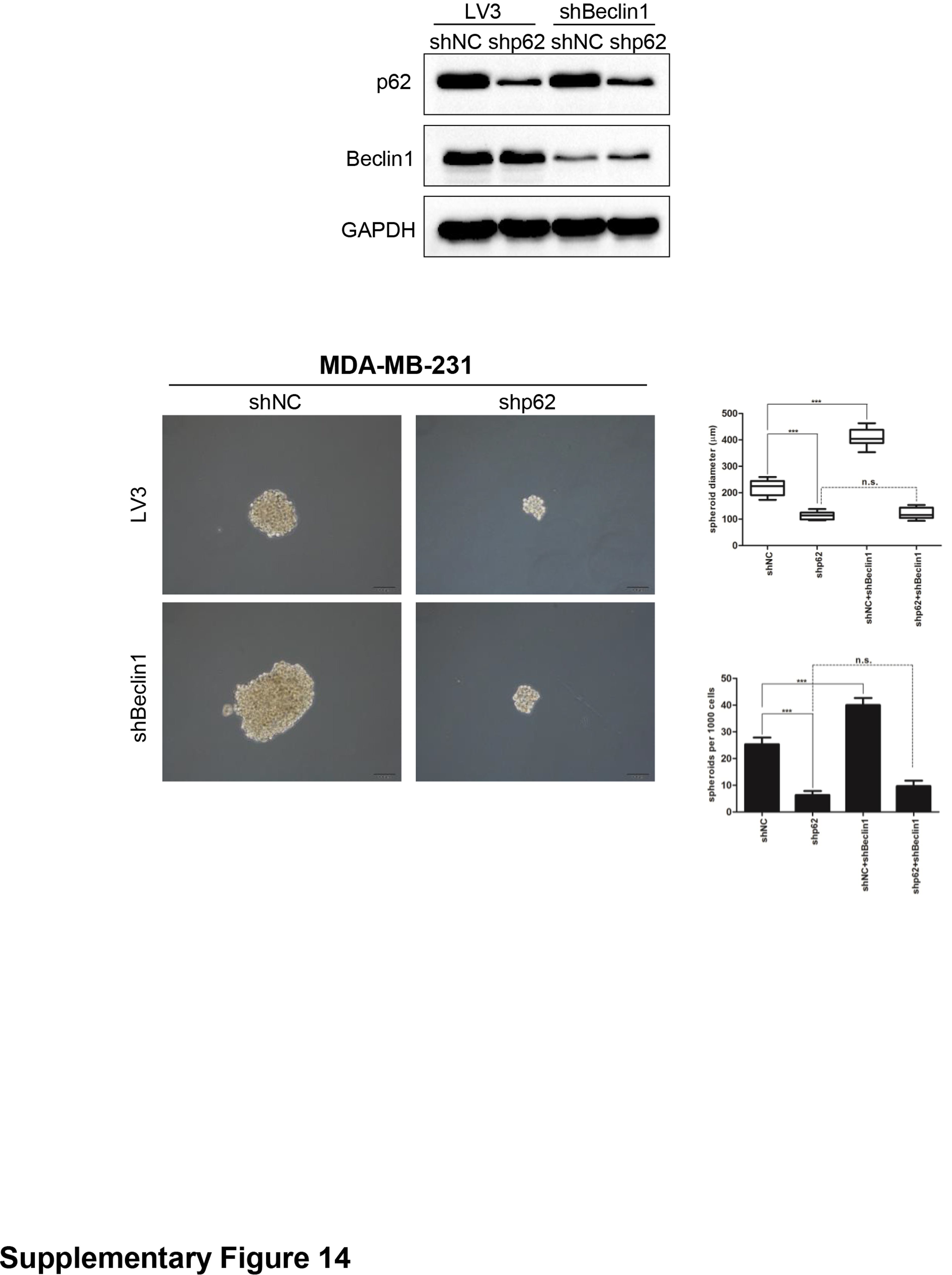

Supplement: Supplementary Figure 14 [file onc2016202x14.tif]

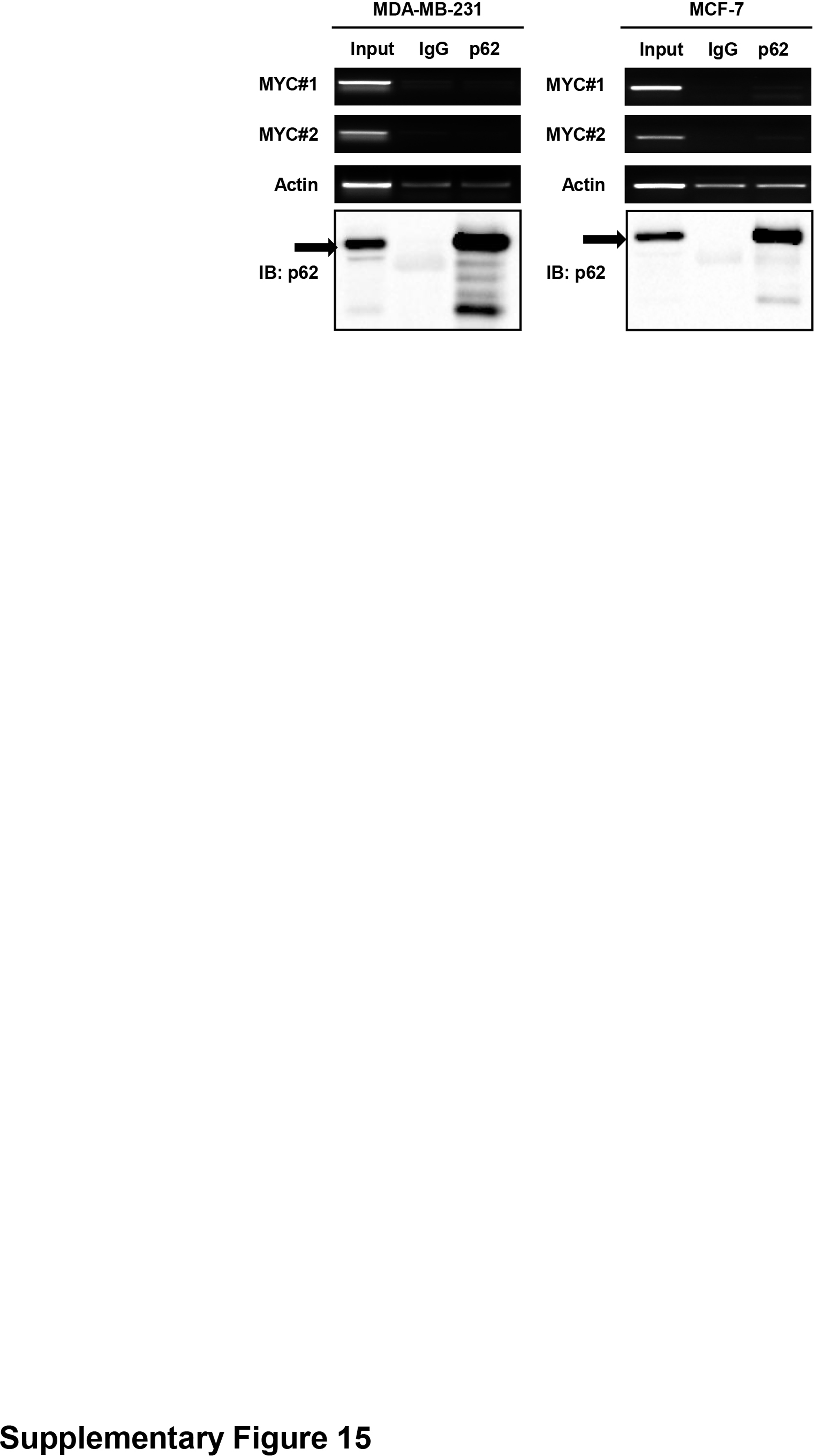

Supplement: Supplementary Figure 15 [file onc2016202x15.tif]

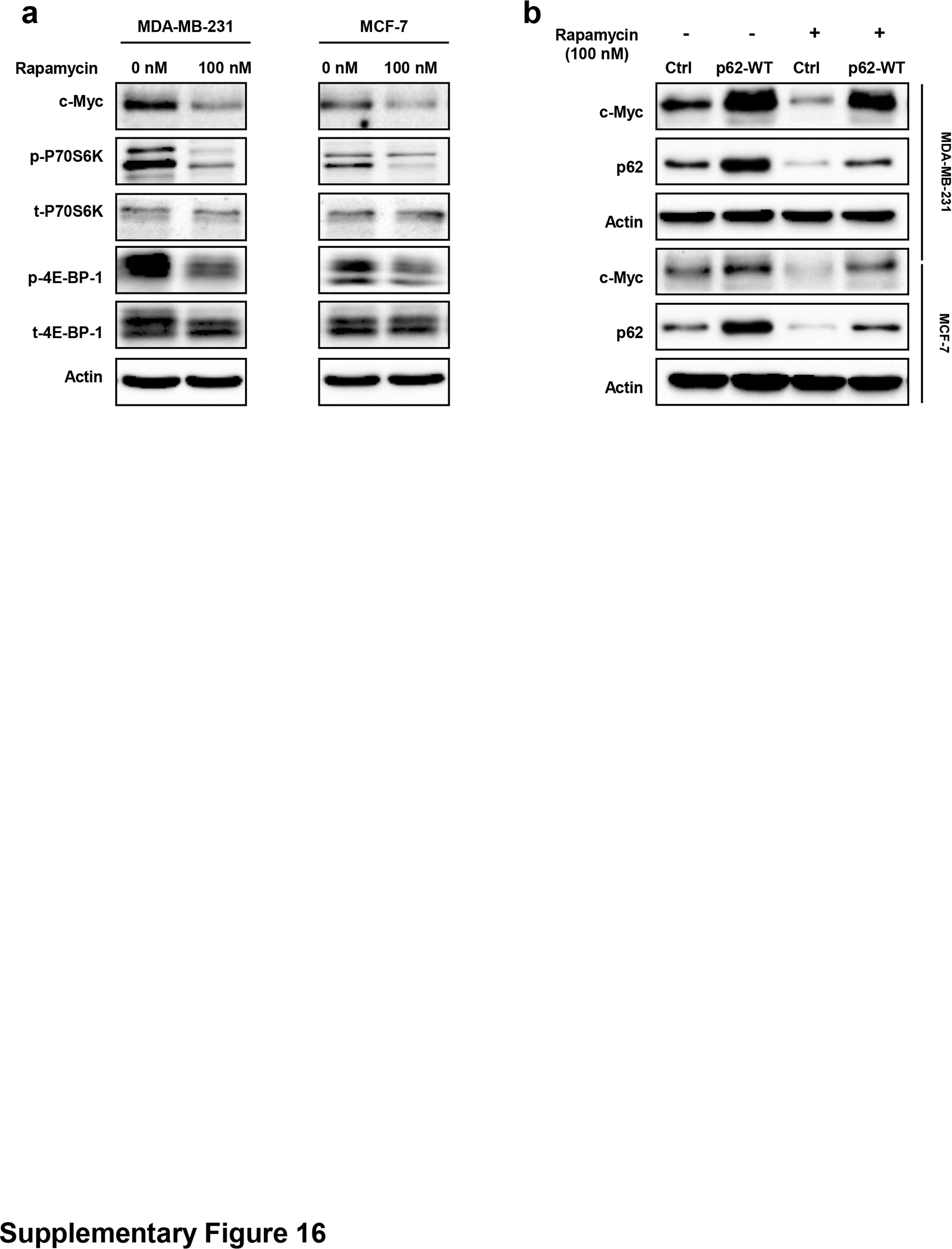

Supplement: Supplementary Figure 16 [file onc2016202x16.tif]
