# Supplementary material for: p62/SQSTM1 enhances breast cancer stem-like properties by stabilizing MYC mRNA
Source: Oncogene. 2016 Jun 27;36(3):304–17. doi: 10.1038/onc.2016.202 (PMC5269535; doi:10.1038/onc.2016.202)
Supplement: Supplementary Information [file onc2016202x17.doc]

**Supplementary Figure legends**

**Supplementary Figure 1 (A)** BCSC-enriched populations were enriched for sphere formation assay. Expression of candidate genes (*p62*, *SOX2*, *NANOG*, *POU5F1*) was analyzed by RT-qPCR. ***P<0.001, two-tailed Student’s t-tests. Error bars represent mean ± SD. **(B)** Efficiencies for knockdown of p62 were tested by Western blot analysis. **(C)** Comparison of plate colony formation numbers and **(D)** mammosphere forming abilities were analyzed following p62 interference. **P<0.01, ***P<0.001. Error bars represent mean ± SD. Scale bars, 100 μm.

**Supplementary Figure 2** p62 expression levels among different breast cancer cell lines were detected by Western blotting assay (left). Actin was loaded was an internal control. Relative abundance was normalized to the MCF-10A cell line (right, the column graph).

**Supplementary Figure 3** Western blotting assay **(A)** and RT-qPCR assay **(B)** were conducted to examine NANOG, SOX2 and POU5F1 (OCT4) expression levels following p62 depletion and overexpression in breast cancer cells. **P<0.01, ***P<0.001. Error bars represent mean ± SD.

**Supplementary Figure 4** Expression intensity of Ki67 was calculated between shNC^#2^ and shp62-2^#2^ xenografts. ***P<0.001. Error bars represent mean ± SD. Detailed method was described in Supplementary Materials and Methods.

**Supplementary Figure 5** Suppression of p62 attenuates the tumor-initiating ability of BCSC-derived tumor xenografts. **(a)** MDA-MB-231-shNC and shp62 cells were immunostained with ALDEFLUOR. The ALDH1-positive subpopulations were isolated by FACS. Inset displays the negative control; cells incubated with DEAB, the specific inhibitor of ALDH, were used to establish the baseline fluorescence of these cells. **(b)** Expression of candidate genes (*p62*, *SOX2*, *NANOG*, *POU5F1*) was analyzed by RT-qPCR. **P<0.01, ***P<0.001, two-tailed Student’s *t*-tests. Error bars represent mean ± SD. **(c) (d)** NOD/SCID mice (n=6) were subcutaneously inoculated with equal number of ALDH1^+^ cells (5×10^4^ cells per mouse). Mice were sacrificed after 6 weeks and tumor volumes monitored as described in Methods and Materials. ***P<0.001.

**Supplementary Figure 6** Gene set enrichment analysis of MYC downstream target genes (FDR≤0.25 and P<0.05 were considered significant.).

**Supplementary Figure 7** Dual luciferase assay was performed in MCF-7 cells as described in Supplementary Methods and Materials. Transcription activity was calculated as the ratio of Firefly luciferase activity (reporter) verses Renilla luciferase activity (control). Error bars represent mean ± SD. Comparison was analyzed by two-tailed Student’s *t*-tests.

**Supplementary Figure 8** MCF-7 cells were transiently transfected with wild-type (WT) p62 construct for 48 hours. Actinomycin D (ActD, 5 μg/ml) was added for indicated time points (0, 30, 60, and 90 minutes) before harvest. MYC mRNA levels were assessed by RT-qPCR analysis. **(a)** Transfection efficiencies of p62 were determined by RT-PCR analysis. **(b)** Relative fold of expression was calculated by normalizing mRNA level to that at 0 minute (without ActD treatment) for both control (Ctrl) and p62-overexpressed (p62-WT) groups. Results are representative of three independent experiments. Error bars represent mean ± SD. **(c)** Half-life of mRNA for each group was predicted according to fold described in **(b)**.

**Supplementary Figure 9** MDA-MB-231 and MCF-7 cells were transfected with let-7a/b mimics for 72 hours. Cells were then harvested and subjected to Western blotting assay. Dicer abundance was used to evaluate the working efficiency of let-7a/b mimics.

**Supplementary Figure 10** Growth rates of breast cancer cells (up to 4^th^ ~ 6^th^ day) were measured by MTT proliferative assay (described in Supplementary Materials and Methods.). Error bars represent mean ± SD. Comparison was analyzed by two-tailed Student’s *t*-tests.

**Supplementary Figure 11 (A)** Cell proliferation was determined by measuring BrdU incorporation using flow cytometry. Error bars represent mean ± SD. n.s.: not significant. **(B)** Cell cycle was analyzed by propidium iodide staining and measured by flow cytometry. Sub G0/G1 phase, S phase and G2/M phase were analyzed using flow cytometry. Error bars represent mean ± SD. n.s.: not significant.

**Supplementary Figure 12** Apoptotic ratio was measured by Annexin-V/PI staining using flow cytometry. Error bars represent mean ± SD. n.s.: no significant.

**Supplementary Figure 13 (A)** Western blotting assay was conducted to detect p62 and LC3 (Ⅰ and Ⅱ) expression levels following p62 depletion and overexpression in breast cancer cells (S: short exposure; L: long exposure). LC3 conversion (Ⅱ/Ⅰ+Ⅱ) was measured to indicate relative autophagy activities. **(B)** 1^st^, 2^nd^ and 3^rd^ generation mammosphere forming abilities were compared. **P<0.01, ***P<0.001. Error bars represent mean ± SD. Scale bars, 100 μm. Autophagic features were assessed by MDC staining (Scale bars, 20 μm).

**Supplementary Figure 14** Efficiencies of shRNA-mediated suppression of Beclin1 in MDA-MB-231-shNC and shp62 group were examined by Western blot analysis. Mammosphere forming ability was compared. ***P<0.001. n.s.: not significant.

**Supplementary Figure 15** RNA immunoprecipitation was performed in MDA-MB-231 cells and MCF-7 cells as described in Supplementary Materials and Methods. MYC mRNA abundance was detected by RT-PCR using 2 different primers (MYC#1, #2). Actin was employed as an internal control. Pulling-down efficiency of p62 was tested by immunoblotting (arrows indicated p62 location).

**Supplementary Figure 16 (a)** MDA-MB-231 cells and MCF-7 cells were treated with Rapamycin (100 nM) and then harvested for Western blotting analysis. Phosphorylation levels of P70S6K and 4E-BP-1 were used to assess drug efficiency. **(b)** MDA-MB-231 cells and MCF-7 cells were pre-treated with 100 nM Rapamycin for 12 hours before p62 ectopic overexpression. Cells were then lysed and subjected to Western blotting analysis.

**Supplementary Materials and Methods**

*Patients and follow-up*

This study comprised 369 female breast cancer patients diagnosed between August 1999 and October 2008 at Sun Yat-sen University Cancer Center. All patients were staged according to the American Joint Committee on Cancer (AJCC) TNM staging system for breast cancer (seventh edition). Paraffin-embedded pathologic specimens were retrieved from the archives of the Department of Pathology of Sun Yat-sen University Cancer Center. The institute Research Medical Ethics Committee of Sun Yat-sen University granted approval for this study. All sample donors gave written informed consent. All patients included in this study had follow-up records for over 5 years. After the completion of therapy, patients were observed at 3 months intervals. Disease free survival (DFS) was defined as the interval from the date of operation to the date of first recurrence. Overall survival (OS) was defined as the period from the date of diagnosis to the date of death.

*Tissue microarray*

Tissue microarray sections were constructed as described previously[^1^](#_ENREF_1). Briefly, representative areas of each specimen were identified on the corresponding slides stained with hematoxylin and eosin. Tissue cylinders with 0.6 mm diameter were punched from each donor tissue block and entered into a recipient paraffin block using a tissue-arraying instrument (Beecher Instruments, Silver Spring, MD, USA). The recipient paraffin block was subsequently cut (5 μm thick), and the slices were transferred with adhesive tape onto coated slides. Then, the slides were dipped in paraffin to prevent oxidation. Each sample was arrayed in triplicate to minimize tissue loss and to overcome tumor heterogeneity.

*Immunohistochemistry Assay*

Immunohistochemical analysis was performed as previously described[^1^](#_ENREF_1). Briefly, the sections were deparaffinnized in xylene, rehydrated and incubated in 3% (v/v) hydrogen peroxide (Sigma-Aldrich, 323381) for 10 minutes to remove the activities of endogenous peroxidases. Antigenic retrieval was processed with sodium citrate. Then the sections were incubated overnight at 4°C with the indicated antibodies in a moist chamber. After incubation with the universal secondary antibody (Solution B; SP-9000, ZSGB-BIO, China), the slides were incubated with streptavidin which was labeled by the horse radish peroxidase (Solution C; SP-9000, ZSGB-BIO, China) for 15 minutes at room temperature. The reaction products were visualized by staining with DAB (3, 3’-Diaminobenzidine; ZSGB-BIO, China). Finally, the sections were counterstained with hematoxylin, dehydrated and mounted. Negative controls were employed by replacing the primary antibody with the non-immune serum immunoglobulin. Staining was assessed and scored by two independent pathologists who were blinded to the clinicopathological characteristics.

The cells were regarded as positive for Ki67 when immunoreactivity was clearly observed in the nucleus. 10 visual fields were randomly chosen under microscope, and positive cell numbers in 100 cells were counted. The average value was represent as the expression intensity of Ki67. Immunohistochemical staining for p62 was evaluated according to intensity and extent. The staining intensity was graded as following: negative (score 0), bordering (score 1), weak (score 2), moderate (score 3) and strong (score 4). The staining extent was also grouped into five parts according to the percentage of high-staining cells in the field: negative (score 0), ≤25% (score 1), 26%-50% (score 2), 51%-75% (score 3) and 76%-100% (score 4). The merged overall score﹥5 was regarded as high staining and those ≤4 were considered as low staining.

*Mammosphere formation assay*

Single-cell suspension was obtained by trypsinization, and clumped cells excluded with a 30 μm sieve. Single cells (1×10^3^ cells/ml) were plated in ultralow attachment 6-well plates. The cells were maintained in DMEM/F12 (Gibco) supplemented with 2% (v/v) B27 (Invitrogen), 20 ng/ml EGF (Sigma-Aldrich), 20 ng/ml basic fibroblast growth factor (bFGF; BD Biosciences), and 4 μg/ml Heparin (Sigma-Aldrich) for 10 days. The mammospheres were counted and photographed using inverted fluorescence microscope (Olympus). The diameters of the mammospheres were calculated with the CellSens Dimension software (Olympus).

*ALDEFLUOR assay and separation of the ALDH positive population by FACS*

The ALDEFLUOR kit (STEMCELL, #01700) was used for isolating the population with a high ALDH enzymatic activity. Cells were suspended in ALDEFLUOR assay buffer containing ALDH substrate (BAAA, 1 μmol/l per 1×10^6^ cells) and incubated for 45 minutes at 37℃. As negative controls, for each sample of cells an aliquot was treated with 50 mmol/L diethylaminobenzaldehyde (DEAB), a specific ALDH inhibitor. The ALDH1-positive subpopulation was isolated by FACS.

*Side population assay*

Fluorescence-activated cell sorting of side population (SP) cells was performed as previously described[^2^](#_ENREF_2). Briefly, MDA-MB-231 cells in the logarithmic growth phase were trypsinized, washed twice with PBS, and counted. Then the cells were resuspended in DMEM with 2% FBS at a final concentration of 1×10^6^ cells/ml and divided into two groups. Group 1 was incubated with the DNA binding dye Hoechst 33342 (Sigma Aldrich) at a final concentration of 5 μg/ml for 90 minutes at 37°C with gentle agitation every 15 minutes. Group 2 was pretreated with 10 μM Fumitremorgin C (FTC) (Sigma Aldrich) at 37°C for 30 minutes, and then incubated with Hoechst 33342 (5 μg/ml) for 90 minutes at 37°C with gentle agitation every 15 minutes. The incubation was carried out in the dark. Then the cells were washed twice with ice-cold PBS and resuspended in HBSS with 2% FBS and 10 mM HEPES. The cell suspension was stored at 4°C while protected from light before FACS, and freshly stained with PI (Sigma Aldrich) at a final concentration of 2 μg/ml. Cell sorting was performed using a fluorescence-activated cell sorter (BD Biosciences). SP and non-SP cells were collected separately and subjected to RT-qPCR analysis subsequently.

*Cell plate colony formation assay*

Log-phased cells (1×10^3^ cells per dish) were plated into 60 mm petri dishes and cultured at 37°C equipped with 5% CO_2_. Cells were fed with fresh growth medium every 3 days. After 14 days of incubation, colonies were fixed with 4% PFA, stained with crystal violet, and counted using Image J software. Each experiment was repeated three times.

*MTT proliferative assay*

MTT proliferative assay was performed as previously described[^3^](#_ENREF_3). Briefly, cells were seeded into 96-well flat bottom plates. At the end of different time points, 20 μl of MTT solution (5 mg/ml, Sigma) was added to each well and cells were incubated at 37℃ for another 4 hours. The absorbance (OD) was measured at 492 nm using a multimode plate reader (Perkin Elmer).

*BrdU incorporation*

Cell proliferation was measured via 5-bromo-2’-deoxyuridine (BrdU) incorporation using a BrdU cell proliferation Detection Kit (KGA319-1; Keygen). Briefly, cells were treated with 30 μM BrdU for 30 minutes at 37℃. Subsequently, the cells were washed with phosphate buffered saline (PBS), trypsinized, and quenched with media. After centrifugation, cells were resuspended in fixation buffer. Fixed cells were stored at 4℃ overnight. The other day, after washing, cells were resuspended in permeation buffer for 2 minutes on ice. Then cells were resuspended in the DNA denaturation solution at 37℃ for 30 minutes. Next, cells were resuspended in 195 μl dyeing buffer, and incubated with 5 μl FITC-BrdU antibody at 4℃ for 30 minutes in darkness. Twenty-thousand cells were analyzed using flow cytometry.

*Cell cycle detection*

PI staining was employed to assess the cell cycle distribution. Briefly, different groups of cells were seeded in 6-well flat bottom, then cells were harvested, washed twice in PBS, and fixed in 75% pre-cold ethanol at 4℃ for 4 h. After RNase A (100 μg/ml) (2158; TaKaRa, Dalian, China) digestion at 37℃ for 30 min, the cells were stained with 50 μg/ml PI (P4170; Sigma) for 15 min at room temperature before analysis with a flow cytometer.

*Apoptosis detection*

Apoptosis was examined using the AnnexinV-FITC/propidium iodide (PI) Apoptosis Detection Kit (KGA107; Keygen). Briefly, cells were seeded in 6-well flat bottom, then harvested, washed twice in phosphate buffer solution (PBS), and stained with AnnexinV-FITC/PI according to the manufacturer’s instructions. The resulting fluorescence was detected by a flow cytometer (BD Accuri C6).

*Lentivirus preparation*

HEK293T cells were used for packaging lentivirus with the 2^nd^ generation packaging system plasmid psPAX2 (Addgene) and pMD2.G (Addgene). Lentiviruses were concentrated by ultracentrifugation, and viral titer determined by serial dilutions. For infection with the lentivirus, infected cells were selected with Puromycin (2 μg/ml) (Sigma-Aldrich).

*Animal studies*

For xenograft transplantation assay, equal amounts (1×10^6^/100 μl in PBS containing 50% Matrigel) of single cells were subcutaneously inoculated into NOD/SCID female mice (4-6 weeks of age). Tumor formation was monitored for 8 weeks. The tumor volumes were determined by the method described previously[^4^](#_ENREF_4). All animal studies were approved by the Institute Animal Care and Use Committee of Dalian Medical University, and carried out in accordance with established institutional guidelines and approved protocols.

Isolated single cell suspensions were prepared from tumor xenografts. Briefly, tumor tissues were mechanically and enzymatically dissociated by incubating at 37°C for 2 hours in collagenase I (2 mg/ml) and filtered through a 40 μm pore filter (BD Biosciences). For limiting dilution assay, equal amounts of cells were serially diluted from 1×10^6^ to 1×10^3^/ 100 μl in PBS containing 50% Matrigel (BD Biosciences), and then subcutaneously injected into NOD/SCID female mice (4-6 weeks of age). Tumor formation was monitored for 8 weeks. Tumor samples were then harvested from euthanized mice. For immunohistochemical analysis, xenografted tumor tissues were fixed with 4% paraformaldehyde, embedded in paraffin and prepared into 5-μm-thick tissue sections. For mRNA and protein expression analysis, xenografted tumor tissues were frozen immediately and stored at liquid nitrogen for subsequent RNA extraction and Western blot.

*RNA extraction, reverse transcription-PCR and real-time quantitative PCR*

Total RNA was extracted by using TRIzol reagent (Life technologies, 15596026). For MYC mRNA decay analysis, cells transfected with p62 or empty vector were treated with 5 μg/ml Actinomycin D (ActD, Sigma Aldrich) for indicated time points (0, 30, 60, 90 minutes, respectively) and then harvested. cDNA was generated by using EasyScript One-Step gDNA Removal and cDNA Synthesis SuperMix Kit (TransGen Biotech, #AE311-03) according to the manufacturer’s instructions. PCR amplification was performed in a 20 μl reaction system by using 2×EasyTaq PCR SuperMix (TransGen Biotech, AS111). Real-time quantitative PCR was performed by using the specific SYBR Select Master Mix (Life technologies, 4472908) in a MX3000p cycler (Stratagene). Changes of mRNA levels were determined by the 2^-△△CT^ method using Actin for internal crossing normalization. Detailed primer sequences for RT-PCR and qPCR were listed in Supplementary Table 1.

For let-7a/b expression analysis, cells were lysed in QIAzol lysis reagent (QIAGEN, 44503618), and microRNA was extracted by using the miRNeasy Mini Kit (QIAGEN, 217004). The cDNA was generated in a single step by using the miScript Reverse Transcription Kit (QIAGEN, 218061) according to the manufacturer’s instructions. Real-time quantitative PCR was performed with the miScript SYBR Green PCR Kit (QIAGEN, 218073) for detection of the let-7 cluster in combination with miScript Primer Assays (QIAGEN, MS00006482, MS00003122, MS00003129, MS00031227, MS00006489, MS00008337). Changes of let-7a/b levels were determined by the 2^-△△CT^ method using RNU6-2 (QIAGEN, MS00033740) for internal crossing normalization.

*Dual-luciferase reporter assay*

For dual-luciferase reporter assay, MDA-MB-231 and MCF-7 cells were seeded into 12-well plates at the confluence of 70%. Cells were transiently cotransfected with pGL3-MYC reporter construct or empty pGL3-basic vector, phRL-TK Renilla control plasmid, and p62 or empty vector using Lipofectamine2000 (Invitrogen). After transfection, luciferase activity was measured using the Dual-Luciferase Reporter Assay System Kit (Promega, E1910) according to the manufacturer’s instructions and quantified on a luninometer (PerkinElmer). Transcription activity was calculated as the ratio of Firely luciferase activity (reporter) v.s. Renilla luciferase activity (control). All data were analyzed from at least three independent experiments, and statistical significance was validated by Student’s t test.

*Western blot analysis*

Samples were lysed on ice in RIPA buffer (50 mM Tris [pH 8.0], 150 mM sodium chloride, 0.5% sodium deoxycholate, 0.1% SDS, and 1% NP-40) supplemented with protease inhibitors (1 mM Na_3_VO_4_, 1 μg/mL leupeptin, and 1 mM PMSF). The protein concentration was determined by the Coomassie brilliant blue dye method. In all, equal amounts of protein per lane were run in 6% to 15% SDS–PAGE gels and subsequently transferred to a nitrocellulose membrane (Millipore) via submerged transfer. After blocking the membrane at room temperature for 1 hour, the membrane was incubated overnight at 4°C with various primary antibodies. After incubation with peroxidase-conjugated secondary antibodies (Thermo Scientific) for 1 hour at room temperature, the signals were visualized using an enhanced chemiluminescence Western blot detection kit (K-12045-D50; Apgbio, Beijing, China) according to the manufacturer’s instructions. The blots were developed using the Bio-Rad Molecular Imager instrument (Bio-Rad, USA). The information of antibodies were listed as follows: Actin (Proteintech, #60008-1), p62 (Santa Cruz, sc-28359), LC3B (Sigma-Aldrich, L7543), c-Myc (Cell Signaling, #5605), Nanog (Cell Signaling, #4903), Sox-2 (Cell Signaling, #3579), Oct-4 (Cell Signaling, #2750), Dicer (Cell Signaling, #3363), Beclin1 (Cell Signaling, #3738).

*Monodansylcadaverine (MDC) staining*

Monodansylcadaverine (MDC, Sigma) was applied to stain autophagic vacuoles. Briefly, cells were fixed in 2% para-formaldehyde-PBS at room temperature for 20 minutes and incubated with the auto-fluorescent dye MDC with a final concentration of 0.05 mM for 10 minutes at 37℃. Cells were then viewed under a fluorescence microscope (Olympus).

*RNA and protein immunoprecipitation*

Cells were transiently overexpressed p62 for 72 hours, then washed and scraped with ice-cold PBS before lysed in ice-cold GLB buffer (10 mM Tris-HCL (PH7.5), 10 mM Nacl, 10 mM EDTA, 0.5% TritonX-100) freshly supplemented with indicated concentrations of Protease Inhibitor Cocktail, DTT and PMSF. Lysates were mixed gently and incubated for 20 minutes on ice, then centrifuged at 12000g for 15 minutes at 4°C. Supernatants of the extractions were taken out 50 ul as Input and the remaining was divided into IgG and p62 IP groups. Non-specific background was removed by rotating at 4°C for 1 hour with additions of Protein G agarose beads (Roche, #11719233001) and 4 M NaCl. Supernatants of IgG and p62 IP groups were incubated with the p62 or IgG attached Protein G agarose beads at 4°C by rotating for 5 hours, respectively. Extensive washing was performed with GLB+ buffer (50 ml GLB supplemented with 1.75 ml 4 M NaCl) containing tRNA (Ambion). RNA was isolated, purified with RNeasy Kit (Qiagen) and subjected to RT-PCR analysis. Protein was eluted with loading buffer by boiling 5 minutes at 100℃ and subjected to immunoblot analysis.

*Statistical analysis*

Clinicopathological characteristics were assessed between two subgroups by the chi-squared test. Cumulative survival probabilities were calculated through the Kaplan-Meier method. Survival rates were compared by log-rank test. Multivariate analyses were performed by the Cox regression model. Variables, such as age, tumor stage, node stage and p62 protein expression were included in the multivariate analysis with enter model. The Kruskal–Wallis test, followed by a Dunn multiple comparison test, was used to perform a statistical comparison with regard to spheroid diameter distribution. The two-tailed Student’s *t*-tests was used to perform a statistical comparison between two groups unless otherwise indicated. Statistical tests were performed using the SPSS software, version 16.0 (SPSS Inc.) or with GraphPad Prism 5.0 (GraphPad Software, Inc.). The level of statistical significance was set at *P < 0.05,* *P <0.01, ***P <0.001.

References

1 Xu J, Wu X, Zhou WH, Liu AW, Wu JB, Deng JY *et al*. Aurora-A identifies early recurrence and poor prognosis and promises a potential therapeutic target in triple negative breast cancer. PLoS One 2013; 8: e56919.

2 Goodell MA, Rosenzweig M, Kim H, Marks DF, DeMaria M, Paradis G *et al*. Dye efflux studies suggest that hematopoietic stem cells expressing low or undetectable levels of CD34 antigen exist in multiple species. Nat Med 1997; 3: 1337-1345.

3 Xu LZ, Long ZJ, Peng F, Liu Y, Xu J, Wang C *et al*. Aurora kinase a suppresses metabolic stress-induced autophagic cell death by activating mTOR signaling in breast cancer cells. Oncotarget 2014; 5: 7498-7511.

4 Zheng FM, Long ZJ, Hou ZJ, Luo Y, Xu LZ, Xia JL *et al*. A novel small molecule aurora kinase inhibitor attenuates breast tumor-initiating cells and overcomes drug resistance. Mol Cancer Ther 2014; 13: 1991-2003.
